# Senotherapeutic peptide treatment reduces biological age and senescence burden in human skin models

**DOI:** 10.1038/s41514-023-00109-1

**Published:** 2023-05-22

**Authors:** Alessandra Zonari, Lear E. Brace, Kallie Al-Katib, William F. Porto, Daniel Foyt, Mylieneth Guiang, Edgar Andres Ochoa Cruz, Bailey Marshall, Melissa Gentz, Gabriela Rapozo Guimarães, Octavio L. Franco, Carolina R. Oliveira, Mariana Boroni, Juliana L. Carvalho

**Affiliations:** 1 OneSkin, Inc., San Francisco, CA USA; 2 Genomic Sciences and Biotechnology Program, Catholic University of Brasilia, Brasília, 70790-160 DF Brazil; 3 Porto Reports, Brasília, 72236-011 DF Brazil; 4 Bioinformatics and Computational Biology Lab, Brazilian National Cancer Institute (INCA), Rio de Janeiro, 20231-050 RJ Brazil; 5 Centre of Proteomic Analyses and Biochemistry, Genomic Sciences and Biotechnology Program, Catholic University of Brasilia, Brasilia, 70790-160 DF Brazil; 6 S-Inova Biotech, Biotechnology Program, Catholic University Dom Bosco, Campo Grande, 79117-010 MS Brazil; 7 Molecular Pathology Program, University of Brasilia, Brasilia, 70.910-900 DF Brazil; 8 Interdisciplinary Biosciences Laboratory, Faculty of Medicine, University of Brasília, Brasília, 70.910-900 DF Brazil

**Keywords:** Senescence, Drug discovery

## Abstract

Cellular senescence is known to play a role in age-related skin function deterioration which potentially influences longevity. Here, a two-step phenotypic screening was performed to identify senotherapeutic peptides, leading to the identification of Peptide (Pep) 14. Pep 14 effectively decreased human dermal fibroblast senescence burden induced by Hutchinson-Gilford Progeria Syndrome (HGPS), chronological aging, ultraviolet-B radiation (UVB), and etoposide treatment, without inducing significant toxicity. Pep 14 functions via modulation of PP2A, an understudied holoenzyme that promotes genomic stability and is involved in DNA repair and senescence pathways. At the single-cell level, Pep 14 modulates genes that prevent senescence progression by arresting the cell cycle and enhancing DNA repair, which consequently reduce the number of cells progressing to late senescence. When applied on aged ex vivo skin, Pep 14 promoted a healthy skin phenotype with structural and molecular resemblance to young ex vivo skin, decreased the expression of senescence markers, including SASP, and reduced the DNA methylation age. In summary, this work shows the safe reduction of the biological age of ex vivo human skins by a senomorphic peptide.

## Introduction

Aging is a complex process associated with tissue dysfunction, functional deterioration, and increased rates of degenerative diseases^1^. In the skin, aging is influenced by many underlying intrinsic and extrinsic processes, such as time (i.e. chronological age), genetic background, and environmental conditions^2–4^. The understanding of these and other events that influence skin aging and function have paved the way for the comprehension of the impacts of age-related skin deterioration on overall organismal health.

The presence and accumulation of aging-associated senescent cells in the skin has long been observed^5–7^. Senescent cells were previously considered to only be byproducts of tissue aging, but more recent studies demonstrate them to be active inducers of aging and dysfunction^1,8,9^, justifying the need for the development of senotherapeutic approaches to treat aging-related diseases. Considering that senescent cells play an active role in skin aging, a mosaic model has been proposed to explain age-related skin deterioration, in which senescent cells accumulate. This actively promotes further tissue dysfunction, affecting the local tissue microenvironment via senescence-associated secretory phenotype (SASP). SASP includes several proinflammatory molecules, as well as matrix metalloproteinases and growth factors^3,10^, and it can compromise epidermal stem cell renewal^11^, extracellular matrix (ECM) deposition^12^, exacerbate melanin synthesis^13^, among others. In this scenario, the selective elimination or suppression of senescent cell populations might effectively interrupt such feed-forward dynamics in the skin. This strategy has been tested using animal models in other tissue niches, such as the hematopoietic system^14^, bone^15^, hair, and in the overall organism^16^. Despite this, clearing certain populations of senescent cells has led to unwanted outcomes such as defects in acute wound healing^17^, strengthening the need for caution and the value of identifying alternatives to senolytics. In this sense, senomorphics, compounds that modulate or reverse the phenotypes of senescent cells, have been described as alternatives^18^. Currently, only one study demonstrates evidence supporting the hypothesis that senotherapeutic strategies may be effective at promoting human skin rejuvenation and documented the positive effects of topical Rapamycin treatment in the skin^19^. However, the knowledge regarding cellular senescence and SASP modulation has not been definitively translated into effective interventions for skin aging.

Here, we screened novel peptides according to their potential to significantly reduce cellular senescence levels in a Hutchinson-Gilford Progeria Syndrome (HGPS) model of cellular aging. One peptide obtained from the screening and optimization process, Pep 14, was chosen for further experiments. Pep 14 decreased the level of cellular senescence in many experimental models and prevented and blocked paracrine induction of cellular senescence, acting as a senomorphic molecule. In single-cell experiments and pseudotime analysis, Pep 14 was shown to likely suppress senescence by preventing late senescent cell accumulation. The underlying mechanism of action is likely via the modulation of a subunit of PP2A, a lesser explored target in the longevity field. When used in 2D and 3D aged skin models, Pep 14 promoted tissue rejuvenation. In human skin, Pep 14 promoted increased epidermal thickness and improved numerous skin health markers, leading to superior results compared to Retinol, the gold-standard for “anti-aging” skin treatments. Taken together, we suggest that senomorphic molecules may contribute to the development of next generation interventions specifically designed to act on the underlying causes of aging and promote skin tissue health on the molecular and cellular level.

## Results

### Screening of a peptide library identifies Pep 14 as a new senotherapeutic compound

**Fig. 1:**
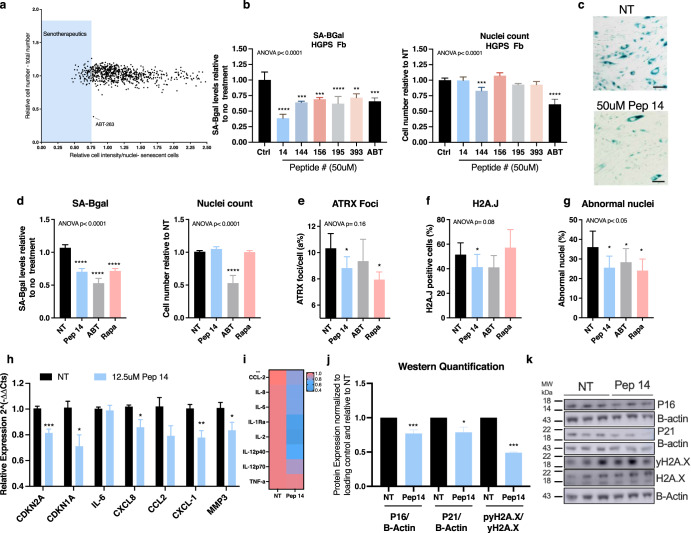
Screening of a peptide library identifies Pep 14 as a new senotherapeutic compound. **a** Screening of the 764-peptide leads according to the senotherapeutic potential. Peptides that reduced the number of senescent HDFs isolated from an HGPS donor by more than 25% after 48 h treatment were considered senotherapeutics and highlighted in the blue shaded area. **b** Relative cellular senescence level and total cell number of HGPS HDFs treated for 48 hours with 50 μM of the top 5 peptides screened in **a**, and 5 μM ABT. **c** Representative SA-BGal stained image of HGPS HDFs treated with 50 μM Pep 14 compared to untreated controls. Scale bar 50 μm. **d** Relative cellular senescence and relative total cell number, **e** mean ATRX foci/cell, **f** mean percentage of H2A.J positive cells, and **g** mean percentage of abnormal nuclei of HGPS HDFs treated with 12.5 μM of Pep14, 5 μM ABT and 100 nM Rapamycin for 48 h. **h** mRNA expression of HGPS fibroblasts treated with 12.5 μM Pep 14 for 48 h. **i** ELISA quantification of SASP components produced by HGPS fibroblasts treated with 12.5 μM Pep 14 for 48 h. **j** Protein expression analysis of P16, P21, and γH2A.x/H2A.x in HGPS fibroblasts treated with 12.5 μM Pep 14 for 48 h. **k** Representative Western Blot analysis of P16, P21 and γH2A.x/H2A.x protein expression. Data are shown as mean ± SD and are representative of 3–4 independent experiments in triplicate and were analyzed using one-way ANOVA and multiple comparisons or Student’s *t* test two-tailed. *p < 0.05; **p < 0.01; ***p < 0.001; ****p < 0.0001, compared to untreated controls (NT).

A library of 164 non-cytotoxic peptides from previous studies^20^ was synthesized and screened for the presence of senotherapeutic molecules by measuring senescence-associated beta-galactosidase staining (SA-BGal) of highly senescent primary human dermal fibroblasts (HDF) from an HGPS donor. The HGPS-derived HDF cell population presented a senescent profile, including approximately 50% SA-BGal+ cells, the accumulation of nuclear ATRX foci^21^, and high expression of genes related to cellular senescence. Such characteristics were similar to the ones presented by HDF obtained from healthy older-aged donors (Supplementary Fig. 1). The initial screening was executed and peptides that reduced the number of senescent cells by more than 25% were considered senotherapeutic (Supplementary Fig. 2a). The top performing peptides were further validated (Supplementary Fig. 2b) and used as reference amino acid sequences to generate 764 leads. The modified peptide library was then assessed for toxicity and senotherapeutic potential (Fig. 1a). Five peptides displaying the lowest cytotoxicity and highest senotherapeutic potential were validated (Fig. 1b, c), and Pep 14 was selected for further investigation. We observed that the relative number of SA-BGal+ cells decreased with numerous concentrations of Pep 14, which were not toxic to senescent and non-senescent cells up to 50 μM (Supplementary Fig. 2c). In this and other sets of experiments, reference molecules were included, namely: i. the senolytic ABT-263, which has been shown to selectively eliminate senescent cells and rejuvenate tissues like the bone marrow in rodents^14^ and also to treat senescence induced photoaging pigmentation in vitro^22^; and ii. the senomorphic Rapamycin, which is a long-studied molecule affecting mTOR/nutrient signaling and has recently been shown to topically decrease P16 (*CDKN2A)* levels in aging skin^19^.

HGPS-HDFs treated with Pep 14 displayed a significant decrease in senescence markers, including SA-BGal^5^, the recently described skin cellular senescence marker histone variant H2A.J^23^, the accumulation of nuclear ATRX foci, as well as abnormal nuclei^24^, in addition to mRNA and protein levels of genes related to aging and SASP (Fig. 1d–i). Western blotting confirmed the reduction of senescence markers P16 and P21, as well as DNA damage marker γH2A.x with Pep 14 treatment (Fig. 1j, k).

We then assessed whether Pep 14 could also reduce the senescence levels of HDF obtained from healthy, older-aged donors. Increased SA-BGal+ cells were observed with increased age of HDF donors, as was the accumulation of nuclear ATRX foci (Supplementary Fig. 1a–c), a marker of senescent cells^21^. The mRNA levels of *CDKN2A* and *CDKN1A* were increased with donor age, while *MKI67* mRNA levels decreased with HDF donor age (Supplementary Fig. 1d–f). Aged HDF (71–90ys) treated with 1.56 μM to 50 μM Pep 14 led to a statistically significant decrease in the number of SA-BGal+ cells (Supplementary Fig. 2d). Based on these results 12.5 μM Pep 14 was chosen for further experiments. At this dose, we tested peptide uptake by treated cells using a fluorescence tagged version and uptake peaked at 24 h, with peptide distribution as mainly cytoplasmic (Supplementary Fig. 3). Peptide 14 was also shown to be a safe molecule. No toxicity, carcinogenesis, or chromosomal instability potential were observed by the Ames test, micronucleus assay, or karyotyping analysis^25^. Moreover, long-term monitoring of dermal fibroblasts led to no significant changes in proliferation and morphology (data not shown).

### Pep 14 protects HDFs from different inducers of cellular senescence

**Fig. 2:**
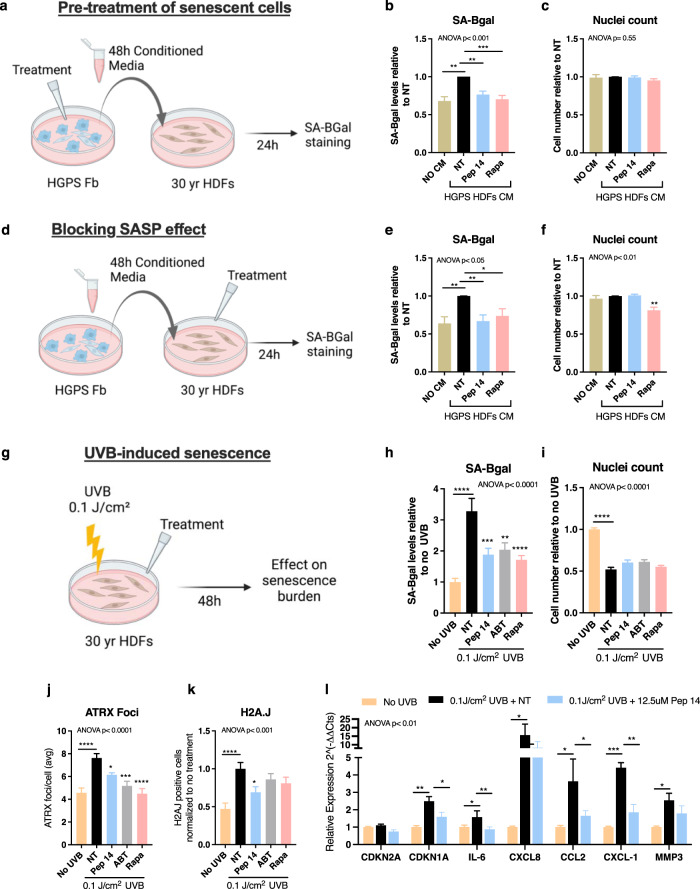
Pep 14 protects cells from different cellular senescence inducers. **a** HDFs isolated from HGPS patients were treated with either 12.5 μM Pep 14 or 100 nM Rapamycin for 48 h. Then the CM was collected and used to treat HDFs isolated from a 30 yr healthy donor for 24 h. Relative cellular senescence (**b**) and relative total cell number (**c**) of samples processed as described in **a**. **d** Alternatively, HDFs isolated from HGPS patients had CM collected and used to treat HDFs isolated from a 30 yr healthy donor for 24 h in combination with either 12.5 μM Pep 14 or 100 nM Rapa treatment. Relative cellular senescence (**e**) and relative total cell number (**f**) of samples processed as described in **d**. **g** HDFs isolated from a 30 yr healthy donor were exposed to 0.1 J/cm^2^ of UVB radiation and treated for 48 h with 12.5 μM Pep 14, 5 μM ABT and 100 nM Rapamycin. **h** Relative cellular senescence, **i** relative total cell number, **j** mean ATRX foci/cell, and **k** mean percentage of H2A.J positive cells of HDFs after 48 h treatment. **l** mRNA expression of HDFs without treatment, exposed to 0.1 J/cm^2^ UVB and exposed to UVB followed by treatment with 12.5 μM Pep 14. Data were normalized to the mRNA expression of untreated HDFs not exposed to UVB. Data are shown as mean ± SD and are representative of ≥3 independent experiments in triplicate. *p < 0.05; **p < 0.01; ***p < 0.001; ****p < 0.0001, compared to untreated control, according to one-way ANOVA and multiple comparisons. Schemes created with Biorender.com.

Senescent cells have been shown to induce senescence markers in a paracrine manner^26^. To determine the effects of Pep 14 over the paracrine induction of the senescence marker SA-BGal, we cultured HGPS-HDFs to generate a pro-senescence conditioned media (CM). The potential of Pep 14 to prevent the generation of a senescence-inducing milieu by senescent cells was determined by adding the peptide directly to media on HGPS-HDF cells for 48 h. The CM derived from the treated HGPS-HDF was collected and incubated with 30 yr HDF for 24 h (Fig. 2a). We also assessed whether Pep 14 was able to block the paracrine senescence induction of SA-BGal expression, by collecting the CM from HGPS-HDF and treating 30 yr HDF for 24 h with both the CM and Pep 14 (Fig. 2d). Pep 14 was used at 12.5 μM, and 100 nM Rapamycin was included as a reference senomorphic molecule^27^. In both experiments, CM from HGPS-HDFs significantly increased SA-BGal staining of 30 yr HDF and Pep 14 was able to both significantly reduce the SASP production (Fig. 2b, c) and block the paracrine induction of the senescence marker SA-BGal (Fig. 2e, f), as determined by a significant reduction in SA-BGal staining that was also observed with Rapamycin.

Given that cellular senescence may be caused by different stimuli, we tested whether Pep 14 could support skin cells to withstand UVB irradiation and etoposide treatment. We exposed HDF from a 30 yr donor to 0.1 J/cm^2^ UVB and immediately treated with either Pep 14, ABT-263, or Rapamycin for 48 h (Fig. 2g). UVB-exposed HDFs treated with Pep 14 presented decreased SA-BGal staining/nuclei, reduced nuclear ATRX foci, and H2A.J staining, as well as a reduction in mRNA levels of genes related to cellular senescence and SASP (Fig. 2h, l) compared to UVB-dosed controls without treatment. Similar observations were made with Etoposide-induced senescent cells treated with Pep 14, which presented lower levels of senescence markers (Supplementary Fig. 4). These data support Pep 14 as capable of protecting dermal cells from multiple forms of extrinsic stressors that contribute to senescence and aging.

### Pep 14 acts as a senotherapeutic agent by regulating aging-related pathways

**Fig. 3:**
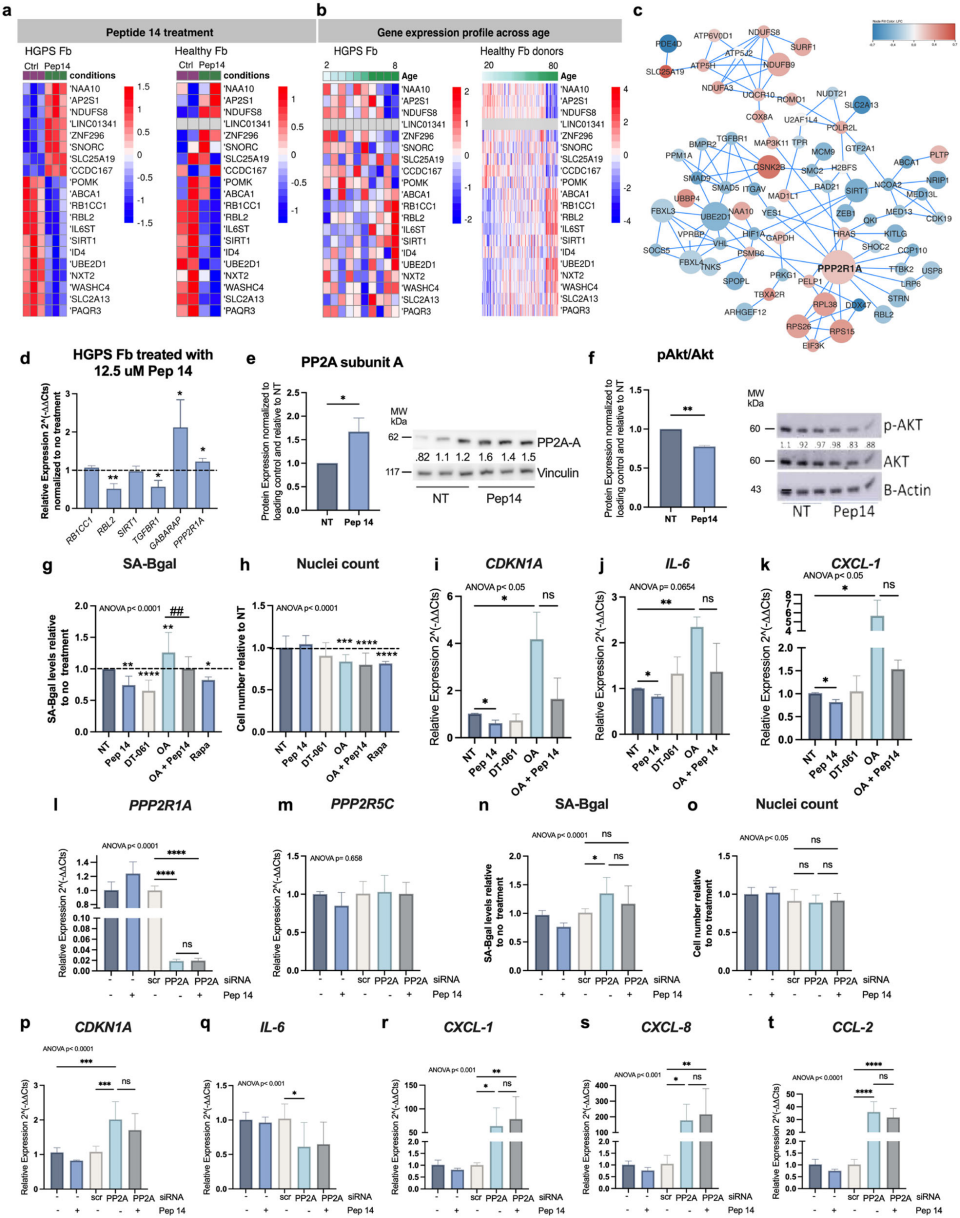
Pep 14 acts as a senotherapeutic agent by regulating aging-related pathways. **a** Heat maps showing the expression pattern of top 20 genes modulated by Pep 14 treatment (12.5 μM) in healthy and HGPS HDFs. Samples were hierarchically clustered using distance as 1 − Pearson correlation coefficient. **b** Heat maps of HDFs samples derived from HGPS patient and healthy donors sorted according to the donor’s age. Color codes in all heat maps represent RNA-seq normalized pseudocounts in log2 scale after row-wise z-score transformation. **c** Protein-protein interaction (PPI) map of genes modulated by Pep 14. Network interaction was built based on the String database. Size of genes represent the number of connections and color their LFC (blue and red for genes down- and up-regulated, respectively). **d** qRT-PCR analysis of some of the top 20 genes modulated by 12.5 μM Pep 14 treatment in HDFs obtained from HGPS. Protein expression analysis of PP2A subunit A (**e**) and pAkt/Akt (**f**) in HGPS HDFs treated with 12.5 μM Pep 14 for 48 h. Relative cellular senescence (**g**) and relative total cell number (**h**) of HGPS HDFs treated with 12.5 μM of Pep 14, 7.5 μM DT-061, 7.5 nM OA and 100 nM Rapamycin for 48 h. mRNA expression of *CDKN1A* (**i**), *IL-6* (**j**), and *CXCL-1* (**k**) of HGPS HDFs treated with 12.5 μM of Pep 14, 7.5 μM DT-061, 7.5 nM OA and 100 nM Rapamycin for 48 h. qRT-PCR analysis of *PPP2R1A* (**l**) and *PPP2R5C* (**m**) in HDFs transfected with scramble (scr) or PP2A siRNA, treated or not with 12.5 μM of Pep 14 for 48 h. Relative cellular senescence (**n**) and relative total cell number (**o**) of control (scr) or PP2A siRNA-transfected HDFs, treated or not with Pep 14. qRT-PCR analysis of *CDKN1A* (**p**), *IL-6* (**q**), *CXCL1* (**r**), *CXCL-8* (**s**), and *CCL-2* (**t**) in HDFs transfected with control (scr) or PP2A siRNA, treated or not with Pep 14. Data are shown as mean ± SD and are representative of 3 independent experiments. Ns: *p* > 0.05, *p < 0.05; **p < 0.01, ***p < 0.001, ****p < 0.0001, compared to untreated control, according to one-way ANOVA and multiple comparisons or Student’s *t* test two-tailed.

To understand the peptide’s underlying molecular mechanism of action, we incubated healthy and HGPS-HDFs with Pep 14 for 48 h and processed the samples for bulk RNA-Seq analysis (Supplementary Table 1; Supplementary Fig. 5). We first checked differences in basal gene expression of the top 20 genes (*p* < 0.05, Supplementary Table 2) that were modulated in HGPS-HDFs by Pep 14 treatment and then verified how they were modulated in healthy-HDFs upon treatment (Fig. 3a). To investigate whether the genes modulated by Pep 14 were associated with aging, we used publicly available HDF RNA-seq datasets of 133 healthy (1–94 years) and 10 HGPS donors (2–8 years)^28^. The heat maps showed that the top 20 genes modulated by Pep 14 generally reflected similar expression pattern changes during aging (Fig. 3a), and that Pep 14 treatment promoted a “younger” gene expression signature (Fig. 3b). Interestingly, gene enrichment analysis of the top 20 genes modulated by the peptide treatment displayed pathways associated with cellular senescence, longevity, and FoxO signaling, among others (Supplementary Table 3).

A larger signature consisting of the top 89 genes modulated by Pep 14 (*p* < 0.1; Supplementary Fig. 6A) and Rapamycin (Supplementary Fig. 6b) in HGPS HDFs was also evaluated and compared to age-related gene expression changes (Supplementary Fig. 6c). Most of the genes in the extended signature were also shown to reflect similar expression pattern changes during aging (Supplementary Fig. 6c). Pathway enrichment analysis using the extended signature identified additional pathways including endocytosis, TGF-beta, Th17 cell differentiation, and FoxO signaling (Supplementary Table 4). Interestingly, the genes associated with the Cellular Senescence pathway, *RBL2 (*LogFoldChange - LFC = −0.3, *p* = 0.04), *SIRT1 (*LFC = −0.43, *p* = 0.04), and *TGFBR1 (*LFC = −0.52, *p* = 0.05) also take part in the FoxO signaling. In fact, Pep 14 was found to modulate several genes associated with both Cellular Senescence and FoxO Signaling pathways. Since modulation of FoxO Signaling is also associated with Rapamycin treatment, we sought to check whether the Rapamycin treatment in HGPS samples altered gene expression in the same direction as Pep 14. Only three genes presented similar mRNA expression alterations (Supplementary Fig. 6a, b), indicating that the mechanism of action of Pep 14 and Rapamycin are distinct.

To further understand the mechanism of action of Pep 14, we searched for similar proteins and consensus small molecule gene expression signatures. Interestingly, Pep 14 is not highly similar to any known proteins, but modulates the expression of a small set of genes, including *NRIP1*, that are commonly modulated by YM-155 and Curcumin, two molecules previously shown to reduce senescence^29,30^ (Supplementary Fig. 6d).

As our RNA-seq analysis highlighted the influence of Pep 14 on FoxO signaling, longevity, and cellular senescence pathways, we investigated its potential to modulate Akt, a member of the PI3K/Akt/mTOR pathway that integrates nutrient, stress, and energy signals to control cell growth, metabolism, and senescence^31^, among others^32^. By investigating the connectivity of proteins encoded by 89 genes modulated by Pep 14, we identified *PPP2R1A*, a scaffold regulatory subunit of protein phosphatase 2 (PP2A) (Fig. 3c, Supplementary Table 4), as an important hub in the network and upstream modulator of Akt. qRT-PCR was utilized to validate RNA-seq results and showed a significant reduction in *RBL2* and *TGFBR1*, as well as a significant increase in *GABARAP* and *PPP2R1A* gene expression with Pep 14 treatment in HGPS-HDFs (Fig. 3d).

PPP2R1A is a PP2A subunit and was predicted to interact with at least 14 other proteins coded by the genes in the graph, such as the kinase *YES1*, the transcription factors *RBL2,* FoxO, and the proteasomal subunit *PSMB6*. Pep 14 treatment induced a significant increase in *PPP2R1A* gene levels and protein levels of the PP2A subunit A (Fig. 3d, e), in addition to a significant reduction in phosphorylated Ser473 of Akt (Fig. 3f), providing indications for further elucidation of the mechanism of action of Pep 14.

Since PP2A has not yet been fully explored for cellular senescence modulation, we tested whether the treatment of HGPS-HDFs with a PP2A inhibitor, Okadaic acid (OA), and activator, DT-061, would result in the modulation of cellular senescence markers. Strikingly, while DT-061 treatment resulted in SA-BGal+ cell reduction, OA treatment induced a significant increase in cellular senescence (SA-BGal, *CDKN1A*) and SASP markers (*IL6* and *CXCL1*) (Fig. 3g–k). Strengthening the notion that PP2A is central for the Pep 14 mechanism of action, Pep 14 treatment was capable of rescuing the senescence induction effect of OA (Fig. 3g).

The relevance of PP2A in the Pep 14 mechanism of action was further confirmed by RNAi experiments. Our data showed that *PP2A* knockdown significantly reduced *PPP2R1A* mRNA levels (Fig. 3l) without altering other PP2A subunit genes, such as *PPP2R5C* (Fig. 3m). The effect of PP2A silencing included a significant increase in SA-BGal+ cell levels (Fig. 3n) without leading to significant alterations in cell number (Fig. 3o), as well as significantly increased mRNA levels of *CDKN1A*, *IL-6*, *CXCL1*, *CXCL-8* and *CCL-2* (Fig. 3p–t). PP2A knockdown also resulted in a loss of Pep 14 capacity to reduce cellular senescence (Fig. 3n). Combined, these results highlight Pep 14 as a senotherapeutic molecule that works via PP2A signaling to reduce cellular senescence accumulation.

### At the single-cell level, Pep 14 suppresses late (deep) senescence and reduces SASP expression in senescent cells

**Fig. 4:**
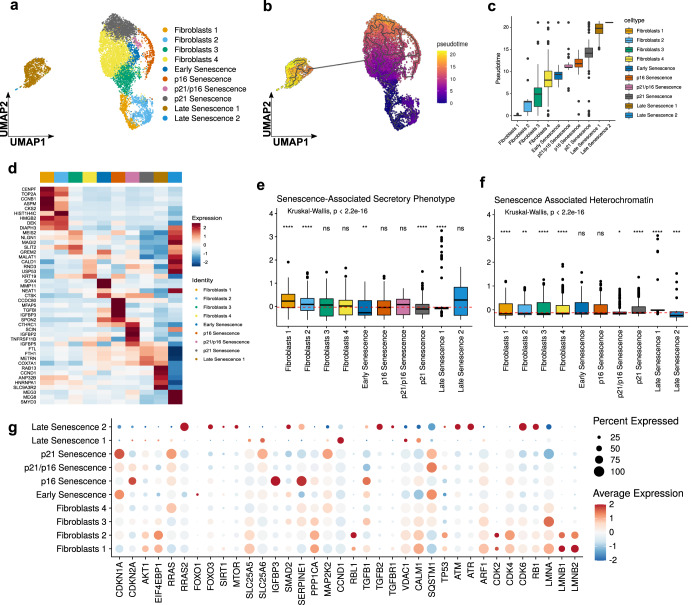
Characterization of HGPS fibroblasts at the single cell level. HGPS fibroblasts were processed for single-cell RNA-Seq. **a** Uniform manifold approximation and projection (UMAP) plot depicting the cell subpopulations identified in the HGPS dermal fibroblasts. The cell subpopulations were named non-senescent Fibroblasts 1–4, Early senescence, P16 senescence, P21 senescence, P21/P16 senescence, late senescence 1, and late senescence 2. **b** Pseudo-time analysis showed the progression of non-senescent fibroblasts to late senescence cells. **c** Pseudotime score for cells grouped by cluster, showing the progression of non-senescent fibroblasts 1–4 to late senescence cells. **d** Heat map showing gene expression markers of HGPS fibroblast subpopulations. Expression levels are depicted in the color code from blue to red. Box plots showing the score based on (**e**) senescence-associated secretory phenotype gene expression levels in each subpopulation and (**f**) senescence-associated heterochromatin gene expression levels in each subpopulation. Dashed lines in red represent the average score. Box indicates the range from 25th to 75th percentile, with whiskers extending to 1.5 times the interquartile range. Outliers are plotted separately, center indicates the median value. **g** Senescence-related gene expression levels in each cluster. For statistical significance, we performed the Kruskall–Wallis test followed by Wilcoxon to compare each of the ten groups against “all” (i.e. base-mean). Ns non-significant. *p < 0.05; **p < 0.01; ***p < 0.001; ****p < 0.0001.

Considering that cellular senescence represents a series of progressive and phenotypically diverse cellular states acquired after the initial growth arrest, we analyzed the senescence progression in HGPS dermal fibroblasts and the effect of Pep 14 treatment at the single-cell transcriptional level. 8616 cells were sequenced and categorized according to their gene expression profile and analyzed by an unsupervised clustering method (Fig. 4a, b). Ten cell subpopulations were identified, including 4 of non-senescent cells (named Fibroblasts 1–4), as well as cells presenting progressive senescence stages. The senescent cell subpopulations were named early senescence, P16 senescence, P21 senescence, P21/P16 senescence, late senescence 1, and late senescence 2. Pseudotime analysis helped understand the progression of the cell subpopulations, from non-senescent to senescent phenotypes (early, P21^+^, P21^+^/P16^+^, and P16^+^ senescence), evolving to late senescent cells (late senescence 1 and 2) (Fig. 4b, c). Furthermore, the analysis helped identify important genes that are modulated during such a transition, such as *B2M* and *TGFB1* (Supplementary Fig. 7).

Each cell subpopulation was characterized by specific transcripts (Fig. 4d) and enriched in different cell cycle phases. Late senescence cells were characterized by a lower number of transcripts (Supplementary Fig. 8a–d), as well as high expression of SASP-related genes, senescence-associated heterochromatin genes (Fig. 4e, f), and general senescence markers (Fig. 4g).

**Fig. 5:**
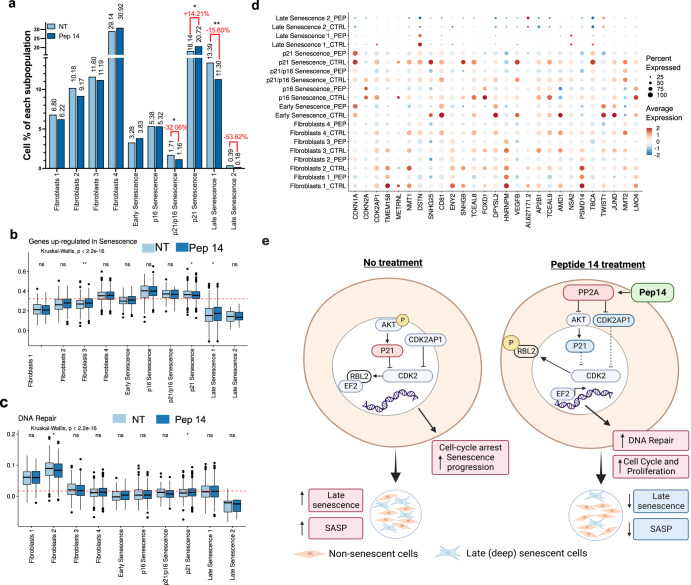
Peptide 14 treatment prevents late senescence cell accumulation. Peptide 14 and untreated HGPS fibroblasts were processed for sc-RNASeq. **a** Frequency of each HGPS cell subpopulation before and after peptide treatment. Differences in frequency were compared using Chi-square test, *p < 0.05; **p < 0.01. Data is shown as mean ± SD. Boxplots showing scores based on (**b**) senescence-related and (**c**) DNA repair-related genes. Dashed lines in red represent the average score. Box indicates the range from 25th to 75th percentile, with whiskers extending to 1.5 times the interquartile range. Outliers are plotted separately, center indicates the median value. Differences between peptide treatment and control were compared using the Wilcoxon test, *p < 0.05; **p < 0.01. **d** Expression levels of the genes that were significantly differentially modulated by Pep 14 treatment, in each cluster. **e** Proposed model for how Pep 14 reduces senescence burden. In the absence of the peptide, CDK2 is inhibited by CDK2AP1 and phosphorylated AKT, favoring the interaction of RBL2 and the transcription factor EF2. Upon Pep 14 treatment, PP2A complex is stabilized, favoring the reduction of CDK2AP1 expression and AKT phosphorylation, leading to CDK2 activation and further phosphorylation of RBL2, liberating EF2 to exert its transcription factor function. Scheme created with BioRender.com.

According to the single cell analysis, Pep 14 treatment significantly increased the number of P21 senescence cells in the population, while reducing P21/P16 senescence by 32.06%, and late senescent cells 1 by 15.60%. Although not statistically significant, late senescence 2 was also reduced by 53.84% (going from 0.39% to 0.18% of total cell population) (Fig. 5a). The reduction of late senescence cells, which presented high levels of SASP-related mRNA expression (Fig. 4e) corroborates the results shown in Fig. 1I and it suggests that peptide treatment suppresses SASP burden by reducing SASP-secreting cells rather than by reducing mRNA levels of SASP genes in all cell subpopulations.

Although Pep 14 increased the number of P21 senescent cells, the cells in this subpopulation had a reduction in senescence-associated gene expression (Fig. 5b), an effect which seems to involve DNA repair activation, since DNA repair genes were significantly increased in such subpopulation (Fig. 5c). Furthermore, Pep 14 treatment consistently modulated senescent-associated genes related to cell cycle progression and epigenetic regulation (Fig. 5d). More specifically, Pep 14 treatment consistently reduced *CDK2AP1* mRNA levels in all cell subpopulations, a gene that modulates CDK2^33^, that together with PPA2 regulates the cell cycle. The long non-coding RNA SNHG25 was also consistently downregulated. This gene regulates HDAC, therefore playing a relevant role in epigenetic modulation^34^.

Of note, Pep 14 treatment did not promote any significant alteration in the expression of genes related to apoptosis and autophagy (Supplementary Fig. 8e, f), corroborating the fact that Pep 14 treatment does not significantly alter nuclei counting in treated samples (Figs. 1b, d, 2c, f, i, and 3h, o). Such observation suggests that the peptide is not a senolytic molecule or that it acts by modulating autophagy. Therefore, taken together, bulk and single-cell RNA sequencing analysis suggests that Pep 14 acts by modulating PP2A and CDKN2AP1 in early-stage senescent cells, supporting DNA repair and preventing those cells from becoming late senescent cells (Fig. 5e).

### Pep 14 reduces skin biological age

To further validate the application of a senomorphic molecule for skin aging, we assessed whether Pep 14 treatment would effectively decrease cellular senescence in more complex skin models. We investigated skin aging using excised human ex vivo skin samples and also replicated skin aging in vitro by building 3D skin equivalents using primary cells derived from older-aged donors.

**Fig. 6:**
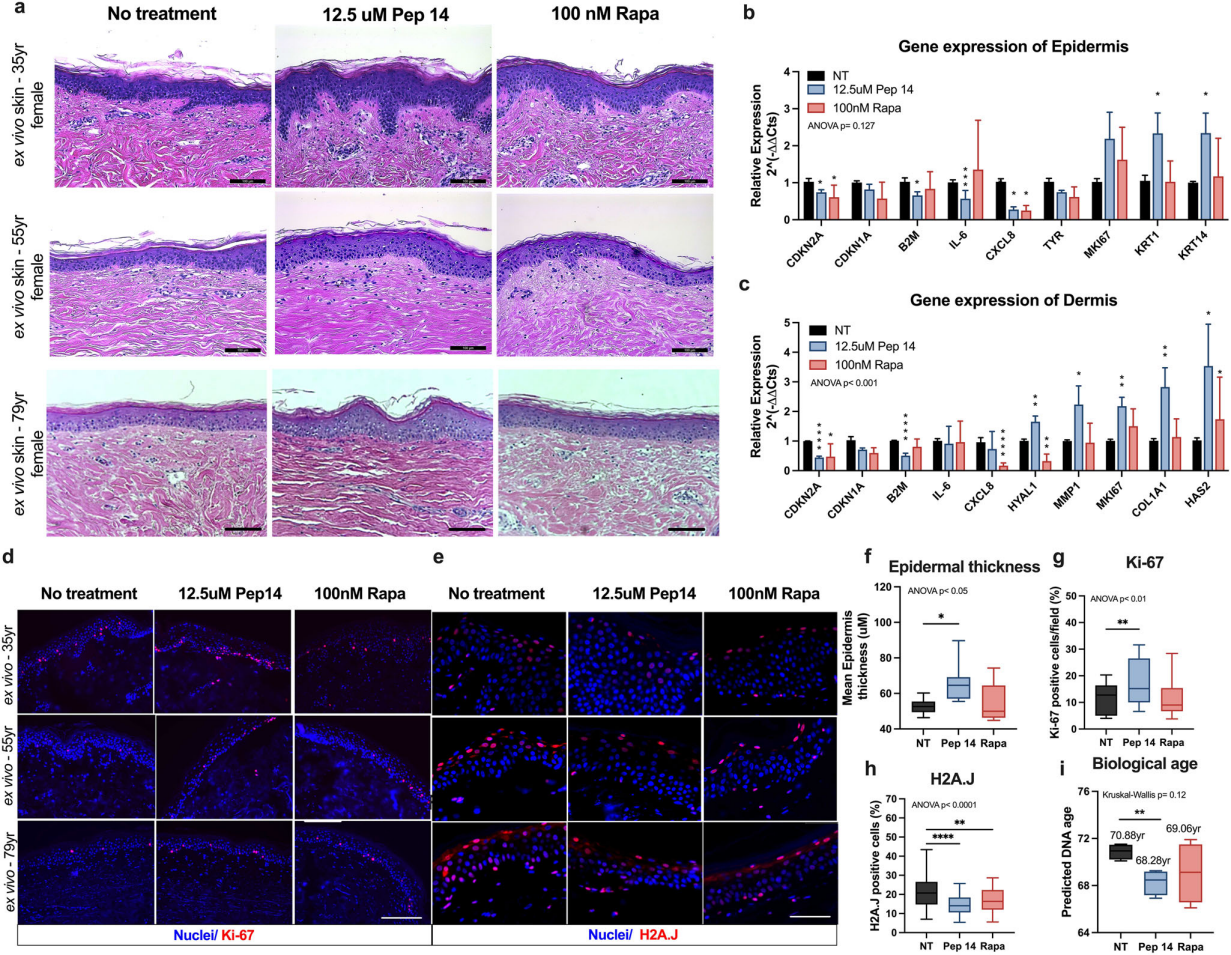
Pep 14 reduces skin biological age. **a** Representative Hematoxylin and Eosin staining of histological sections of ex vivo skin samples from 35, 55, and 79 yr donors maintained in basal media (no treatment) or treated with 12.5 μM Pep 14 and 100 nM Rapamycin (Rapa), added in the tissue culture media for 5 days, scale bar 100 μm. mRNA expression of epidermal (**b**) and dermal (**c**) layers of samples (35, 55 and 79 yr) treated for 5 days. Fluorescence microscopy images (**d**) and quantification (**g**) of ex vivo skin (35, 55, and 79 yr), 5 days after treatment, stained for Ki-67. Fluorescence microscopy images (**e**) and quantification (**h**) of ex vivo skin (35, 55, and 79 yr), 5 days after treatment, stained for H2A.J. Scale bar corresponds to 100 µm. **f** Epidermal thickness analysis of ex vivo skin samples (35, 55 and 79 yr) after 5 days of treatment. **i** DNA methylation age calculated using the Skin-Specific DNA Methylation Clock (MolClock) of ex vivo skin samples (79 yr, female) maintained in basal media (NT), or treated with 12.5 μM Pep 14 or 100 nM Rapamycin (Rapa), added in the media for 5 days. Graph bar data are shown as mean ± SD. Boxplot data are shown as median (center line) and quartiles (1st and 3rd) and the minimum and maximum by the whiskers. Data are representative of 3 independent experiments in triplicate. *p < 0.05; **p < 0.01; ***p < 0.001; ****p < 0.0001, compared to untreated control (NT), according to one-way ANOVA and multiple comparisons or Student’s *t* test two-tailed. For graph **i**, one experiment was performed with 4 biological replicates, statistical analysis was done using Kruskal–Wallis and Wilcoxon test.

Compared to Rapamycin, Pep 14 promoted the maintenance of the overall structure of ex vivo skins of donors with different age ranges (35–79 years old) when added to culture media, leading to thicker epidermis, whereas Rapamycin treatment resulted in a more disorganized epidermal layer, and failed to increase the thickness of the epidermis (Fig. 6a, f). The mRNA expression of Pep 14-treated epidermis from ex vivo skin samples exhibited a significant decrease in the aging markers *CDKN2A* and *B2M*, the SASP marker *CXCL8*, the pigmentation-related gene *TYR*, and a trend towards decreased SASP-related gene *IL6* expression, in addition to a significant increase in *KRT1* (a marker of keratinocyte terminal differentiation) and *KRT14* (a marker of non-differentiated, proliferative keratinocytes) (Fig. 6b). Rapamycin induced a similar decrease in *CDKN2A*, *CXCL8* and *TYR* expression, and a trend towards increased *IL6* expression (Fig. 6b). In the dermis, Pep 14 treatment promoted a significant reduction in *CDKN2A* and *B2M* gene expression, as well as higher expression of the cell proliferation marker *MKI67* and extracellular matrix components *HYAL1*, *MMP1*, *COL1A1* and *HAS2*. Rapamycin treatment induced *CDKN2A*, *CXCL8* and *HYAL1* reduction, and increased *HAS2* (Fig. 6c). Ki-67 alterations was validated at the protein level (Fig. 6d, g). Protein levels of H2A.J were also assessed in the skins, and were reduced by both Pep 14 and Rapamycin treatment (Fig. 6e, h).

We repeated many of these experiments in 3D skin equivalents and first confirmed the aged phenotype of equivalents built with cells derived from elder donors by assessing the overall structure, quality and mRNA expression of the samples, which varied according to the cell donor age (Supplementary Fig. 9). Interestingly, peptide treatment of 3D skin equivalents (Supplementary Fig. 10) led to similar results as compared to ex vivo skin samples (Fig. 6).

Since Pep 14 was observed to reduce cellular senescence in human skin models when added to the culture media (Fig. 6; Supplementary Fig. 10), we investigated whether a topical Pep 14 treatment would also result in improved molecular health in ex vivo skin samples. We created a formulation that supported the penetration of up to 2% of the peptide into the dermis (Supplementary Table 5). The concentration of Pep 14 in the formulation was optimized to 0.01% w/v. Topical Retinol was used as an “anti-aging” control, due to its widespread and decades-long use in the field^35–37^. The overall observations were similar to those made using in-media treatment. Pep 14 treatment improved histological score, reduced mRNA levels of senescence and inflammation markers while increasing keratin and collagen expression, and showed improved cell proliferation (Supplementary Fig. 11).

Most importantly, compared to Rapamycin, Pep 14 treatment of ex vivo skin samples significantly improved numerous markers of skin health and cellular senescence, which reflected its capacity to significantly reduce the skin equivalent DNA methylation age by an average of 2.6 years (*p* < 0.01), as assessed by the MolClock^4^ (Fig. 6h). Similarly, a reduction in the biological age of skins treated with Pep 14 was also detected using other published molecular clocks^38–41^ (Supplementary Fig. 12). Taken together, Pep 14 was shown to promote a younger skin profile histologically, decreased the expression of senescence and SASP-related genes, and showed to be a senomorphic peptide that safely reduces the biological age of skin.

## Discussion

Aging is associated with compromised tissue function and loss of resilience and regeneration upon damage. The role of cellular senescence in local and systemic aging has been previously described^1^ and recognized in the skin^7,42^ with the accumulation of senescent cells compromising skin function. As a few recently published studies demonstrate, reducing senescence burden improves skin function^43,44^. Nevertheless, despite the ease in which human skin cells are collected, the straight-forward construction of skin equivalents in vitro, the increased safety of topical senotherapeutic treatments (which potentially incurs lower risk of systemic toxicity), and the importance of skin aging in overall organismal health^45^, the skin has been largely overlooked as a translatable model in the longevity field.

In the present study, we implemented a screening platform based on human skin cells derived from HGPS donors. Despite obvious differences compared to chronologically aged counterparts, HGPS also share relevant processes to natural aging, such as progerin accumulation, telomere attrition, downregulation of DNA repair and chromatin organization, and upregulation of ERK, mTOR, GH-IGF1, MAPK, TGFβ signaling pathways, in addition to mitochondrial dysfunction, citing a few^46^. Furthermore, we validated our findings using 3D skin equivalents and skin tissue fragments, which faithfully recapitulates skin aging in vitro, therefore increasing the chances of finding molecules with translational benefits^47^. We screened almost 1000 peptides for those acting as a senomorphic instead of a senolytic modulator, of which Pep 14 stood out as a safe molecule, as shown here and also as already published by our group^25^. Our approach resulted in the description of a synthetic peptide that acts through an under-explored mechanism of cellular senescence and longevity modulation.

Pep 14 reduced cellular senescence in numerous senescence-inducing experimental models, which recapitulate both intrinsic and extrinsic stimuli. Treatment with the putative senotherapeutic peptide diminished senescence and inflammation markers by supporting cellular resilience and DNA repair blocking its effects, while enhancing cellular renewal.

Our RNASeq analysis, validated using qRT-PCR and western blotting, showed that Pep 14 acts through the modulation of PP2A/AKT signaling and likely downstream through Rbl2 (p130). PP2A is a serine/threonine phosphatase that regulates several signaling pathways related to cell proliferation, apoptosis and genome stability, being an active target of investigation in the cancer field^48^. Furthermore, PP2A disruption has been related not only to cancer incidence, but also other age-related/aggravated diseases, such as cardiovascular diseases^49^ and neurodegeneration^50^. Its role in longevity is not thoroughly clarified, but PP2A subunit disruption was shown to reduce lifespan in knockout flies^51^ and *C. elegans*^52^.

Currently, PP2A is an under-explored target for cellular senescence modulation. Nevertheless, as shown here, PP2A modulation controls cellular senescence: PP2A inhibition being sufficient to induce cellular senescence, while its activation reduces senescence markers. In this sense, Pep 14 seems to modulate cellular senescence by activating one of PP2A subunits or stabilizing the PP2A holoenzyme, which in turn modulates Akt phosphorylation, indirectly influencing RBL2/E2F. As evidence that Pep 14 modulates PP2A, the peptide rescued cellular senescence induced by OA-induced PP2A inhibition and gene silencing by RNAi. Since PP2A is a heterotrimeric enzyme that may be composed of numerous subunits^53^, further investigation is necessary to determine specific PP2A isoforms and subunits directly involved in cellular senescence modulation by Pep 14 and their roles in related pathways.

At the single cell level, it was possible to shed light into the diversity of senescent and non-senescent cell subpopulations existing within HGPS primary fibroblast cultures. Interestingly, according to pseudotime analysis, Pep 14 treatment significantly increased the amount of non-senescent/early senescence cells, while reducing the number of late-senescence cells, by supporting DNA repair and preventing such cells from entering late senescence. This is another piece of evidence showing, once again, that Pep 14 does not act as a senolytic molecule, failing to induce apoptosis and autophagy-related mRNA expression in all cell subpopulations.

According to the sc-RNASeq, the peptide acts by consistently reducing the mRNA expression of *CDK2AP1* in all cell subpopulations, leading to the activation of CDK2, probably through PP2A stabilization. This ultimately activates CDK2, leading to cell cycle arrest to promote DNA repair, preventing pre-senescent cells to progress to late-senescent cells and accumulate in the population, therefore reducing SASP-secreting cells.

The translational application of Pep 14 to the skin was assessed by investigating the effect of a topical formulation in ex vivo skins. The topical Pep 14 treatment was compared to Retinol, since retinoids are considered reference “anti-aging” active ingredients in the cosmetic industry^35–37,54–58^. Strikingly, Retinol treatment of ex vivo skin samples promoted the peeling effect of the stratum corneum, but also a significant increase in *CDKN2A* mRNA expression in both the epidermal and dermal layers. Furthermore, Retinol induced a significant increase in *CXCL-8* mRNA expression, which is likely associated with the irritation commonly caused by this ingredient. Alternatively, topical Pep 14 increased epidermal thickness, decreased senescence markers, maintained lower levels of inflammation, and induced cell proliferation and collagen expression, leading to a healthier skin profile. Remarkably, 5-day peptide treatment also promoted a significant reduction of an average 2.6 years in the biological age of the tissues, which was not observed with Rapamycin, a molecule that has been shown to reduce cellular senescence, as measured by P16 levels^19^. Such observations were accompanied by an increase in functional health parameters of 3D skin equivalents and ex vivo skins, including an improvement on the morphological tissue organization which resembled a younger skin profile.

While reduction in biological age has been shown several times in reprogrammed samples^4,59,60^, age reversal of tissues is a more challenging task^61,62^. Therefore, the present study stands out as being the first demonstration of how a senotherapeutic peptide rapidly rejuvenated ex vivo skin tissues in only 5 days, as confirmed by different epigenetic DNAm clocks. Since the present study is limited to in vitro human skin cells and equivalents and involved different concentrations of peptide (ranging from 1 to 50 μM) being optimized in different experimental systems, future studies involving human subjects must be executed to define the best peptide dose and determine the benefits of the proposed peptide treatment for the skin.

Taken together, our data demonstrate that Pep 14 is a senotherapeutic compound that supports DNA repair, prevents SASP by preventing cells from progressing to late stages of cellular senescence, therefore improving skin health, and significantly reducing the skin biological age. By using a screening platform based on the cellular senescence phenotype and skin equivalent models, we showed that it is possible to determine novel therapeutic peptides that are able to both reduce cellular senescence accumulation and ameliorate signs of skin aging.

## Methods

### Peptide library construction

A peptide library containing 164 short amino acid peptides from a previous work^20^. The library was synthesized using the SPOT methodology with a functionality of 100 nmol (Kinexus, Canada). The peptides were diluted in water overnight, under agitation, and then diluted in culture media to a working solution of 50 μM.

### Amino acid scanning

The four lead peptides from the peptide library were subjected to an amino acid scanning, where each position was replaced by each one of proteinogenic amino acid residues, generating variants with a point mutation. The new library consisted of 764 new peptides that were synthesized using the SPOT methodology (Kinexus, Canada).

### Chemical synthesis of peptides

The top hit peptides selected (Pep 14, 144, 156, 195, and 393) from the screening and the fluorescence labeled peptide (5FAM-PEG2-Pep 14) were purchased from CPC Scientific Inc. (USA), which synthesized the peptide by solid phase (Fmoc) on a Rink amide resin, with >95% purity, in the form of acetate salt. The molecular mass was confirmed by mass spectral analysis and purity by the RP-HPLC chromatogram.

### Cell culture

Human primary dermal fibroblast cell lines were obtained from The Progeria Research Foundation (PRF) Cell and Tissue Bank. The HGPS cell lines was HGADFN178 (female, 6ys 11 mos). The PRF Cell and Tissue Bank is Institutional Review Board (IRB) approved by the Rhode Island Hospital Committee on the Protection of Human Subjects, Federal Wide Assurance FWA00001230, study CMTT#0146-09.

Healthy normal human dermal fibroblasts and human keratinocytes were either purchased from Coriell Institute for Medical Research (Camden, NJ), MatTek Life Science (Ashland, MA), or isolated from ex vivo human skin explants obtained from ZenBio (Research Triangle, NC). ZenBio complies with ethical regulations and is IRB approved by Pearl IRB, fully accredited by the Association for the Accreditation of Human Research Protection Program Inc. (AAHRPP). A signed informed consent was obtained for all participants.

The cells purchased from Coriell Institute for Medical Research included HDF 71 yr (AG05811, female, arm, white), HDF 84 yr (AG11725, female, arm, white) and HDF 90 yr (AG08712, female, arm, white).

Cells purchased from MatTek Life Science included HDF 60 yr (F13400A, female, african-american), keratinocytes 60 yr (K13400A, female, african-american), neonatal HDFs (F90800, male, foreskin, white), neonatal keratinocytes (K90800A, male, foreskin, white).

All other primary cells were isolated from ex vivo human skin obtained from ZenBio (Research Triangle, NC). All skin samples were from female donors, white, and from the abdominal area. The donor ages included 30, 35, 41, 48, 55 and 79 years. The cell isolation was performed as described by Zonari et al.^63^. These cells have restriction availability. Briefly, the tissue samples were cut into small pieces of 0.5 cm^2^ and incubated in PBS containing dispase (2.5 U/mL, BD Biosciences) overnight at 4 °C. The epidermis was then mechanically separated from the dermis and incubated in 0.5% trypsin-EDTA (Gibco, USA) for 7 min at 37 °C to isolate the keratinocytes. The cells were separated from the remaining tissue using a 100 mm pore size cell strainer (BD Biosciences, USA) and the cell suspension was centrifuged at 290 g for 5 min. Human keratinocytes were seeded at a density of 100,000 cells/cm^2^ in Keratinocyte Serum Free Medium (KSFM) supplemented with Epidermal growth factor and Bovine pituitary extract (Gibco). For the isolation of HDF, the dermis separated from the epidermis was incubated in PBS containing collagenase IA (250 U/mL, Sigma) for 3 h at 37°C. The HDF were separated from the remaining tissue using a 100 mm pore size cell strainer, centrifuged at 300 g for 5 min and seeded at a density of 50,000 cells/cm^2^ in Dulbecco’s Modified Eagle Medium (Invitrogen, Carlsbad, CA), supplemented with 10% v.v. Fetal Bovine Serum (FBS; VWR) and 1% v.v. Penicillin-Streptomycin (Invitrogen).

### 2D screening of senotherapeutic peptides

HGPS HDFs were seeded in 96 well plates at a density of 4 × 10^3^ cells/well at least 6 h prior to treatment. The peptide library was added at a final concentration of 50 μM in DMEM without FBS. After 30 min, 10% FBS f.c. was added. The cells were incubated for 48 h at 37 °C and 5% CO_2_. After incubation, cells were fixed for SA-BGal staining. The screening was repeated 3 independent times. ABT-263 (ApexBio, final concentration: 5 μM) was added as a positive control. Cells without treatment were considered negative control.

### Conditioned media experiments

HGPS HDFs were cultured in 96 well plates for 48 h to create “conditioned media” either in the presence or absence of treatments (experiment 1 Fig. 2A). In the latter condition, treatments were added after (experiment 2 Fig. 2B) moving conditioned media onto 30 yr HDF for 24 h in 96 well plates. SA-BGal staining was then performed as outlined below.

### Cytokine measurements from conditioned media

Condiotioned media from cells seeded on 12 well-plates was collected after 48 h with or without treatment with 12.5 uM of Pep 14. The media was collected from 3 independent experiments in triplicate. The conditioned media was immediately frozen and sent for elisa multiplex analysis by Eve Technologies. The amount of cytokines per sample was normalized by cell number.

### 3D skin model production and treatments

3D skin models were prepared as described by Maria-Engler et al.^64^, with minor modifications. Briefly, collagen I gels containing embedded fibroblasts were seeded with keratinocytes and cultured for 24 h. Then, gels were raised to an air-liquid interface and kept for additional 7 days, to allow epidermal cornification. Treatments were performed by adding the molecule in the culture media or with a topical formulation. In 3D skin equivalent assays, Pep 14 was used at 1 μM, and Rapamycin at 100 nM. Treatment was added to the culture media on day 0 and day 3. Topical Pep 14 treatment consisted of 0.01% Pep 14 in a 15% oil moisturizing emulsion. After 5 days, the samples were harvested and fixed in formalin for histology, or used for RNA isolation.

### 3D skin model quality control

Histology of produced 3D skin models was performed as quality control. To do so, 3D skin models were fixed overnight in 10% Formalin, then embedded in paraffin, sectioned and stained with hematoxylin and eosin (H&E). Quality assessment followed an internal protocol, in which the analysis of several aspects of the obtained models is performed by an experimental group-blinded examiner. Seven different parameters were evaluated by a blinded individual and ranked from 0–4, namely: general organization of cell layers (0 - disorganized layers; 4 - organized layers), stratification of epidermis (0 - low stratification; 4 - high stratification), nucleation of the basal layer (0 - absence of a distinguishable basal layer; 4 - dense nuclei forming a uniform layer of cells), definition of cell limits (0 - hardly visible cell limits; 4 - visible cell limits), adhesion of the basal layer to the dermis (0 - completely detached; 4 - firmly attached), quantity of granular cells (0 - no visible granular layer; 4 - visible and continuous granular layer), and thickness of stratum corneum (0 - absent or thin stratum corneum; 4 - thick stratum corneum). The maximum score is 28.

### Human ex-vivo skin samples and treatments

Skin samples from healthy donors (female, white, ages 35, 55 and 79 years old) that undergo plastic surgery were purchased from ZenBio (Research Triangle, NC). ZenBio complies with ethical regulations and is IRB approved by Pearl IRB, fully accredited by the Association for the Accreditation of Human Research Protection Program Inc. (AAHRPP). A signed informed consent was obtained for all participants. Samples were cultured in an air-liquid interface using DMEM supplemented with 10% FBS and treatments were added either in the media or by topical application using sterile swabs. Treatment was added to the culture media on day 0 and day 3. Topical treatment (described above) was also added on day 0 and day 3. After 5 days from the first treatment, the samples were harvested and fixed in formalin for histology, or used for RNA and DNA isolation. Treatment was performed in 3 separate pieces of skin from each donor.

In some experiments, dermal and epidermal layers were separated prior to processing by incubating the skins overnight in dispase solution 2.5 U/mL (Invitrogen). The epidermis was mechanically separated from the dermis using forceps.

### Epidermal thickness

The epidermal thickness was measured on H&E stained histological sections as described previously^65^. Three tissue sections per group were examined. Three random high-power (20×) images were captured per tissue section, and 5 measurements of the epidermal thickness per field were made. The epidermal thickness is presented as average ± SD in micrometers (um).

### Photoaging experiments with UVB

Cultured fibroblasts were washed with 1× PBS and a small layer left on top. Lids were removed and cells dosed with 0.1 J/cm^2^ UVB (Honle UVASPOT 400/T solar source with Honle UV Meter to determine dosage, Honle, Germany) and media with or without peptide treatment added immediately after dosing. Cells were incubated with treatments for 30 h then either fixed for SA-BGal staining, or harvested for either RNA isolation and qRT-PCR analysis or protein isolation for Western blotting. All experiments were performed in triplicate with at least 3 independent experiments.

### Accelerated aging induction in 2D cultured cells

To induce accelerated aging of fibroblasts, cells were treated for 24 h with 20 μM etoposide (Cell Signaling)^31^. Two days after etoposide removal, the cells were treated for 48 h with 12.5 μM of Pep 14 and the senescence level was determined by SA-βGal staining and normalized to total cell number. The experiment was performed in triplicate in 3 independent experiments.

### HGPS nuclei morphology analysis

To assess the effects of Pep 14 on HGPS HDFs, cells treated for 48 h were visualized by Hoechst staining using the IN Cell Analyzer 2500 at 40× magnification. ImageJ software was used to manually quantify cells that displayed either normal (smoother and oval) or abnormal (blebbing, infoldings, micro nucleoli) nuclear morphology. A total of 45 fields (9 fields/well) of 3 independent experiments were analyzed, which lead to 100–300 nuclei/treatment/experiment.

### PP2A modulation experiments

HGPS HDFs were treated with 7.5 nM Okadaic Acid or 7.5 μM DT-061 for 48 h. Some cells were treated with both 7.5 nM Okadaic Acid and 12.5 μM Pep 14 for 48 h. The senescence level was determined by SA-βGal staining and normalized to total cell number. mRNA and protein were also collected after 48 h for gene expression and protein level analysis. The experiment was repeated 3 independent times in triplicate.

### Western blotting

Cells were homogenized with a Tris-SDS-EDTA Lysis buffer containing protease and phosphatase inhibitors and beta-mercaptoethanol. Samples were boiled and run on SDS-PAGE for western blotting according to standard procedures. Nitrocellulose membranes were probed for p21 (1:1000, CST 2947, Cell Signaling Technology), p16 (1:1000, CST 80772, Cell Signaling Technology), β-Actin (1:200, sc-81178, Santa Cruz Biotechnology), Vinculin (1:200, sc-73614, Santa Cruz Biotechnology), γH2A.X (1:200, sc-517348, Santa Cruz Biotechnology), H2A.X (1:200, sc-517336, Santa Cruz Biotechnology), pAkt (1:1000, CST 9018, Cell Signaling Technology), PP2A-A (1:1000, CST-9780, Cell Signaling Technology), Akt (1:1000, CST 2938, Cell Signaling Technology), and Phospho-Akt1 (Ser473) (D7F10) (1:1000, CST-9018, Cell Signaling Technology). Blots were detected with the Azure Biosystems ECL detection kit (AC2103). All gels derived from the same experiment and were processed in parallel (Supplementary Fig. 13). Quantification of band intensities by densitometry was carried out using ImageJ.

### Senescence associated β-galactosidase staining and quantification

SA-βGal staining was performed using the Senescence -β-Galactosidase Staining Kit (Cell Signaling), following manufacturer’s instructions. Briefly, cells were washed with 1x PBS and fixed for 10–15 min. Then, cells were washed twice with 1× PBS and incubated with SA-βGal Staining Solution overnight at 37 °C in a dry incubator without CO_2_. Cells were then washed and counterstained with 1 μg/mL of Hoechst 33342 (1 μg/mL, Invitrogen, H1399) for 10 min and observed by 20× magnification (6D High Throughput, Nikon). The blue staining was quantified as the mean color intensity compared to the total number of cells using CellProfiler^TM^. Data were presented as SA-βGal staining levels normalized to total cell number.

### ATRX staining and quantification

ATRX was detected by immunofluorescence as described previously^21^. Briefly, the cells were fixed using 4% paraformaldehyde solution for 10 min. Permeabilization was performed for 5 min using 0.1% Triton followed by blocking for 40 min with 0.5% Tween and 1% BSA. The primary antibody against ATRX (1:2000, Santa Cruz Biotechnology, D5 - sc55584) was diluted 1:2000 and incubated overnight at 4 °C. After 3 washes with PBS, cells were incubated at room temperature for 1 h with the secondary antibody Goat Anti-Mouse IgG H&L- Alexa Fluor® 488 (1:250, Abcam, ab150113) and Hoechst 33342 (1 μg/mL, Invitrogen, H1399). Cells were imaged at 40× magnification, using the IN Cell Analyzer 2500 and the IN Cell Developer toolbox (GE Healthcare). The average ATRX foci per cell was defined by the total ATRX foci/total nuclei from a minimum of 150 cells per experimental condition.

### Immunofluorescence microscopy of ex-vivo and 3D skins

Formalin-fixed skin tissues were embedded in paraffin and sectioned to 10 µm thickness. After dewaxing in xylene and rehydration in decreasing concentrations of alcohol, sections were boiled for 20 min in a 10 mM citrate buffer. After cooling, sections were incubated with 1% BSA/5% FBS/0.1% triton for 30 min. Sections were incubated overnight at 4 °C with primary antibodies (1:100 anti-Ki-67 - Abcam - ab16667, 1:800 anti-H2A.J - Active Motive - Cat. No. 61793). After a series of washing, sections were incubated with anti-mouse AlexaFluor-633 (1:250, Thermo Fisher, A21126) or anti-rabbit AlexaFluor-568 secondary antibodies (1:250, Abcam, ab175471) and Hoechst 33342(1 μg/mL, Invitrogen, H1399). Finally, sections were mounted in VECTAshield™ (Vector Laboratories, Burlingame, USA) and images were acquired using a Leica THUNDER Imager. For each donor, 3 technical replicate sections were analyzed and 5 images were acquired per section.

Quantification was performed using ImageJ. For Ki-67 and H2A.J staining, the number of positive-stained nuclei and the total number of nuclei was counted.

### RNA isolation and qRT-PCR

RNA was isolated using the Quick RNA Miniprep kit (Zymo), following manufacturer’s instructions. For qRT-PCR analysis, RNA was quantified using spectrophotometry and 1 μg of RNA of each sample was reverse-transcribed using High-Capacity cDNA Reverse Transcription Kit (Thermo Fisher Scientific), following manufacturer’s instructions. qRT-PCR was performed using PerfeCTa® qRT-PCR ToughMix®, Low ROX™, (QuantaBio) with Taqman (Invitrogen) probes for CDKN2 (P16) (Hs00923894_m1), B2M (Hs00187842_m1), CDKN1A (P21) (Hs00355782_m1), CXCL-8 (Hs00174103_m1), IL-6 (Hs00174131_m1), MKI67 (Hs04260396_g1), Keratin 1 (Hs00196158_m1), Keratin 14 (Hs00265033_m1), TYR (Hs00165976_m1), HAS-2 (Hs00193435_m1), COL1A1 (Hs00164004_m1), HYAL-1 (Hs00201046_m1), MMP-1 (Hs00899658_m1), GABARAP (Hs00925899_g1), FOXO3 (Hs00818121_m1), TGFBR1 (Hs00610320_m1), RBL2 (Hs00180562_m1), RB1CC1 (Hs01089002_m1), SIRT-1 (Hs01009006_m1), PPP2R1A (Hs01026388_m1) and GAPDH (Hs02758991_g1).

### Cell transfection

For siRNA in vitro assays, cells were transfected with 25 nM of specific siRNAs for PPP2R1A gene (siGENOME SMART pool PPP2R1A, Dharmacon, Cat M010259-02-0005, 5 nmol) or a scrambled negative control siRNA (siGENOME Negative non-targeting siRNA, cat D-001206-13-05, 5 nmol) in lipofectamine® RNAiMAX (13778075, ThermoFisher) following manufacture instructions. Cells were transfected for 48 h before treatment with Peptide 14.

### DNA sample acquisition and methylation analysis

Total DNA samples were obtained from human skin biopsy samples (female, white, 79 yr) using the QIAamp DNA Mini Kit (Qiagen) and applied to the human Illumina Infinium EPIC 850 K chip, as described previously^4^. DNA samples included control, 5 days treatment with 12.5 μM Pep 14, and 5 days treatment with 100 nM Rapamycin, 3 independent replicates each.

### Epigenetic molecular clock analysis

Raw methylation files (idat) were converted to beta values using the “minfi” package version 1.32.0^66^ after the quality control and quantile normalization using the function betaqn from the “wateRmelon” package version 1.30.0^67^. Quantile-normalized beta values for all samples were used as input for the Skin-Specific DNAm predictor MolClock^4^ as well as for the function “agep” in the wateRmelon package to calculate the molecular age using the other tools (Horvath^40^, Hannum^39^, Lin^68^, Horvath Skin & Blood^38^, and PhenoAge^41^). Box plots were used to compare age predictions among groups in different datasets.

### RNA-sequencing samples preparation

Total RNA samples were evaluated for integrity by using an Agilent 2100 Bioanalyzer (RNA 6000 Nano Chip Total Eukaryotic RNA Assay, RIN ≥ 9.3). RNA-Seq libraries were constructed using Illumina TruSeq Stranded mRNA Library Prep Kit (Illumina) and sequenced on the Illumina NovaSeq6000 platform in the 100 nt, paired-end configuration.

### RNA-sequencing analysis

For RNA-Seq analysis, both internal and publicly available data sets were used. SRA files from the project SRP144355^28^ were downloaded and converted to fastq files using SRA Toolkit version 2.8.2-1, while internal data was delivered as fastq files. Reads from raw RNA-Seq data (fastq files) were trimmed using the software Trimmomatic version 0.37^69^ with default options and mapped to the human genome (GRCh38 - ENSEMBL release 88) using STAR version 2.5.3a^70^ with default parameters for single unstranded reads as per developer’s manual. Htseq-count version 0.11.1^71^ was used to assign uniquely mapped reads to genes (excluding pseudogenes) according to the annotation in the Homo_sapiens.GRCh38.89.gtf data set (ENSEMBL release 88). Only genes with a minimum mean of 10 mapped reads were considered for further analysis. Read counts were analyzed using the R package DESeq2 version 1.26.0^72^ and libraries were normalized using the estimateSizeFactors function of the package according to the condition evaluated. For fibroblasts, we evaluated the conditions of peptide treatment versus control. Heat maps were constructed using the pheatmap package version 1.0.12 using a regularized Log2-transformed counts-per-million, z-scaled across samples. Genes (rows) were clustered using 1-Pearson correlation coefficient as distance and samples were ordered based on their chronological age, with exception of samples of progeroid fibroblasts (control and treated with Pep 14), that were hierarchically clustered, likewise the genes. The data generated is available in the SRA/NCBI through the BioProject: PRJNA674878.

### Pathway enrichment analysis

Gene lists were further projected onto biological pathways of known biological functions or processes enriched using piNET, which holds a comprehensive library of biological gene/protein sets through Enrichr. The Kyoto Encyclopedia of Genes and Genomes (KEGG) 2019 database was used. P-values were controlled for False Discovery Rate (FDR) using the Bonferroni method.

### Network analysis

The top 300 genes modified by the Pep 14 treatment ranked by *p*-value were used to investigate hub genes considering the protein-protein interaction network provided by the String database^73^. Only high confidence interactions with score cutoff higher than 0.7 were considered. The biggest subnetwork was visualized with Cytoscape (version 3.8)^74^. Genes were colored according to their LFC (Pep 14 versus Control) and their size reflects their connectivity degree.

### Gene perturbation signature

To investigate the mechanism of action of Pep 14, we searched for consensus small molecule signatures that mimicked the extended signature of genes modulated upon treatment. The underlying dataset for the search engine included the top 89 modulated genes upon Pep 14 treatment that were compared to a portion of the LINCS L1000 small molecule expression profiles generated at the Broad Institute by the Connectivity Map team (https://maayanlab.cloud/L1000CDS2/#/index/5f5fb98a77dff10054d75ccc). Signatures were scored according to the overlap between the input genes signature and the signature genes from the database divided by the effective input, which is the length of the intersection between the input genes and the L1000 genes.

### Single-cell cDNA library preparation

Full-length cDNA pools and Illumina libraries were prepared using 10X Genomics kit. HGPS HDFs were treated or not with 12.5 uM of peptide 14. 10000 cells were prepared for sequencing using the 10X Genomics Chromium Next GEM Single Cell 3’ Dual Index. Preparation of the cDNA and library was created according to the manufacturer’s instructions.

### scRNA-seq data acquisition and pre-processing

Reads were aligned to the human reference genome GRCh38 using Cell Ranger (10x Genomics, v.7.0.0), and analyzed in R software version 4.0 through the Seurat package (v4.0)^75^. Control and treatment matrices were individually analyzed, and filtered cell barcodes with <200 expressed genes, >7500 expressed genes, or >5% of reads mapping to mitochondrial RNA were removed in the quality control step.

### Integration and clustering of scRNA-seq

We integrated the pre-processed datasets by using FindIntegrationAnchors and IntegrateData^76^. The Principal Component Analysis (PCA) was performed using the function with default parameters, and 30 principal components were retained for downstream analysis. Based on PCA, dimensionality reduction was performed with the RunUMAP function, and cell clustering was performed using the FindNeighbors with 1:30 dims, followed by the FindClusters function with resolution = 0.3. In order to compare the significant difference between the relative frequencies before and after the treatment with PEP, we apply a Pearson chi-square test.

### Single-cell differential gene expression

Differentially expressed genes (DEG) were calculated by Model-based Analysis of Single-cell Transcriptomics (MAST)^77^ using the FindAllMarkers function using the RNA assay.

### Cell cycle scoring

To characterize the cell cycle for each cell, we calculated G1, G2M, and S scores using the CellCycleScoring function in Seurat.

### Trajectory inference analysis

The Seurat object was converted into a CellDataSet, to use the UMAP generated in the integration as the partition. We next employed Monocle 3^78^ and set the parameter use_partition = FALSE of the function “learn_graph”, in order to create a linear trajectory. To order cells, the root used to infer the pseudotime was the Fibroblast 1 cluster, which was enriched with a G2M score.

### Gene set enrichment analysis and gene signature scores

The top hundred genes identified in the DEG analysis, sorted by the log fold change for each cluster, were selected to perform pathway enrichment by Reactome and KEGG, through the cluster profiler package^79–81^. The gene signature scores were calculated using the AddModuleScore function with the MSigDB following signatures Canonical Pathways (CP) from Reactome: APOPTOSIS, AUTOPHAGY, DNA_REPAIR, SENESCENCE_ASSOCIATED_SECRETORY_PHENOTYPE_SASP, and FORMATION_OF_SENESCENCE_ASSOCIATED_HETEROCHROMATIN_FOCI_SAHF; and from Chemical and Genetic Perturbations (CGP) database the signature named FRIDMAN_SENESCENCE_UP.

### Supplementary information

Supplemental Information

## Data Availability

All sequence data (bulk and single-cell RNA-Seq) generated through this study is available in the SRA/NCBI through the BioProject PRJNA674878, while methylation data can be accessed through GSE230026. Pre-processed scRNA-Seq and methylathion data are also available at https://gitlab.com/lbbc_publications/os1-analysis/-/tree/main/data.

## Code availability

The code for sc-RNA-seq and methylation analysis are available at https://gitlab.com/lbbc_publications/os1-analysis.
