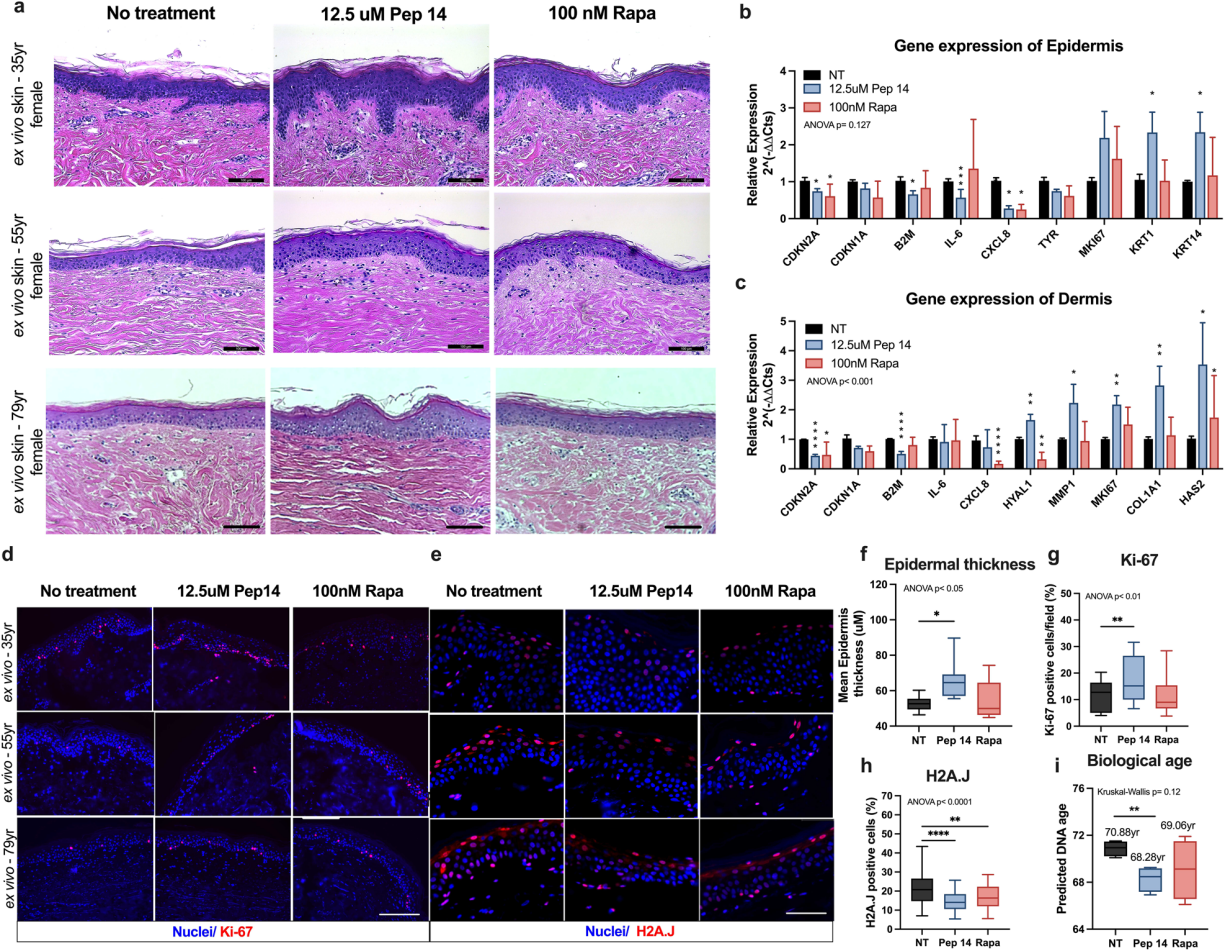# Author Correction: Senotherapeutic peptide treatment reduces biological age and senescence burden in human skin models

**DOI:** 10.1038/s41514-024-00140-w

**Published:** 2024-02-15

**Authors:** Alessandra Zonari, Lear E. Brace, Kallie Al-Katib, William F. Porto, Daniel Foyt, Mylieneth Guiang, Edgar Andres Ochoa Cruz, Bailey Marshall, Melissa Gentz, Gabriela Rapozo Guimarães, Octavio L. Franco, Carolina R. Oliveira, Mariana Boroni, Juliana L. Carvalho

**Affiliations:** 1 OneSkin, Inc., San Francisco, CA USA; 2 Genomic Sciences and Biotechnology Program, Catholic University of Brasilia, Brasília, 70790-160 DF Brazil; 3 Porto Reports, Brasília, 72236-011 DF Brazil; 4 Bioinformatics and Computational Biology Lab, Brazilian National Cancer Institute (INCA), Rio de Janeiro, 20231-050 RJ Brazil; 5 Centre of Proteomic Analyses and Biochemistry, Genomic Sciences and Biotechnology Program, Catholic University of Brasilia, Brasilia, 70790-160 DF Brazil; 6 S-Inova Biotech, Biotechnology Program, Catholic University Dom Bosco, Campo Grande, 79117-010 MS Brazil; 7 Molecular Pathology Program, University of Brasilia, Brasilia, 70.910-900 DF Brazil; 8 Interdisciplinary Biosciences Laboratory, Faculty of Medicine, University of Brasília, Brasília, 70.910-900 DF Brazil

**Keywords:** Senescence, Drug discovery

Correction to: *npj Aging* 10.1038/s41514-023-00109-1, published online 22 May 2023

In this article, the authors found an unintended error in Fig. 6a that resulted in the duplication of the no treatment image for the ex vivo skin of the 79yr female. These images were intended to illustrate the ultrastructural features of the skin; however, they did not impact the main conclusions of the study. These figures should have appeared as shown below. The original article has been corrected.