# Supplementary material for: Senotherapeutic peptide treatment reduces biological age and senescence burden in human skin models
Source: NPJ Aging. 2023 May 22;9(1):10. doi: 10.1038/s41514-023-00109-1 (PMC10203313; doi:10.1038/s41514-023-00109-1)
Supplement: Supplementary file 1 — Supplemental Information [file 41514_2023_109_MOESM1_ESM.pdf]

Supplementary Information

Senotherapeutic peptide treatment reduces biological age and senescence burden in human skin models

Alessandra Zonari [1*], Lear E. Brace [1], Kallie Al-Katib [1], William F. Porto [2,3], Daniel Foyt [1], Mylieneth Guiang [1], Edgar Andres Ochoa Cruz [1], Bailey Marshall [1], Melissa Gentz[1], Gabriela Rapozo Guimarães [4], Octavio L. Franco [2,5-7], Carolina R. Oliveira [1] , Mariana Boroni [1,4], Juliana L. Carvalho [2,8]

1. OneSkin, Inc., San Francisco, California, USA
2. Genomic Sciences and Biotechnology Program, Catholic University of Brasilia, Brasília, 70790-160, DF, Brazil
3. Porto Reports, Brasília, 72236-011, DF, Brazil
4. Bioinformatics and Computational Biology Lab, Brazilian National Cancer Institute (INCA), Rio de Janeiro, 20231-050, RJ, Brazil
5. Centre of Proteomic Analyses and Biochemistry, Genomic Sciences and Biotechnology Program, Catholic University of Brasilia, Brasilia, 70790-160, DF, Brazil
6. S-Inova Biotech, Biotechnology Program, Catholic University Dom Bosco, Campo Grande, 79117-010, MS, Brazil
7. Molecular Pathology Program, University of Brasilia, Brasilia, 70.910-900, DF, Brazil
8. Interdisciplinary Biosciences Laboratory, Faculty of Medicine, University of Brasília, Brasília, 70.910-900, DF, Brazil

*Corresponding author: alessandra@oneskin.co

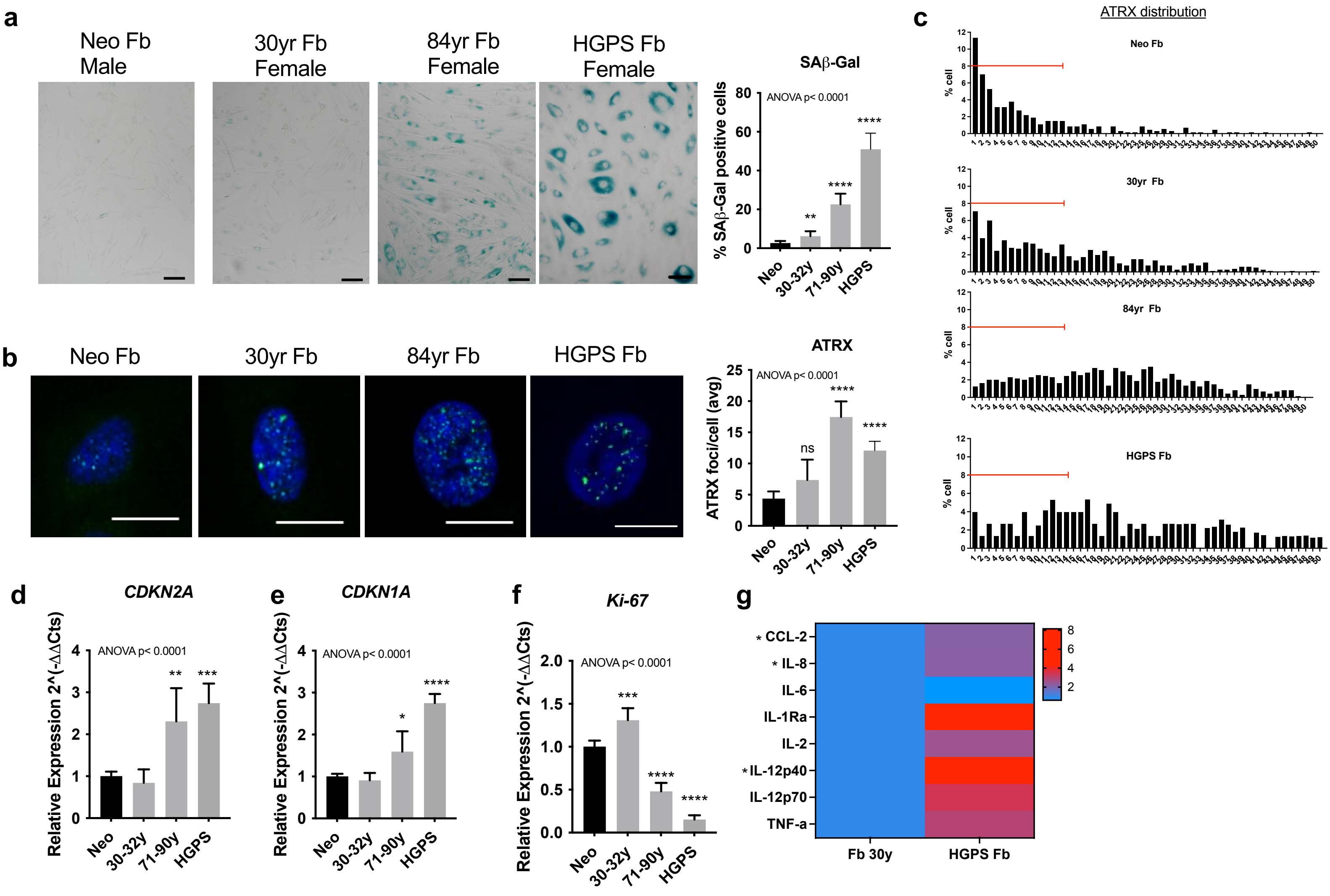

Supplementary Figure 1. Analysis of senescence markers in dermal fibroblasts obtained from healthy donors of different chronological ages, as well as HGPS donors. (a) Representative images of SA-βGal staining of HDFs isolated from neonatal, 30 and 84 year old healthy donors, as well as from a HGPS donor. The percentage of senescent cells was determined in the samples, scale bar 100 μm. (b) Representative ATRX foci staining of human primary fibroblasts isolated from neonatal, 30 and 84 year old healthy donors, as well as from a HGPS donor, with the quantification of the percentage of senescent cells in the samples. Scale bar 20 μm. (c) Representative analysis of the percentage of fibroblasts with specific numbers of ATRX foci/cell in the samples analyzed in (B). mRNA expression of senescence and proliferation markers, CDKN2A (d), CDKN1A (e) and Ki-67 (f). (g) Quantification of SASP components in conditioned medium of fibroblasts derived from a 30 year old healthy donor, as well as from a HGPS donor. **p<0.01; ***p<0.001; ****p<0.0001, according to One-Way ANOVA and multiple comparisons test. Data are shown as mean ± SD, n=3.

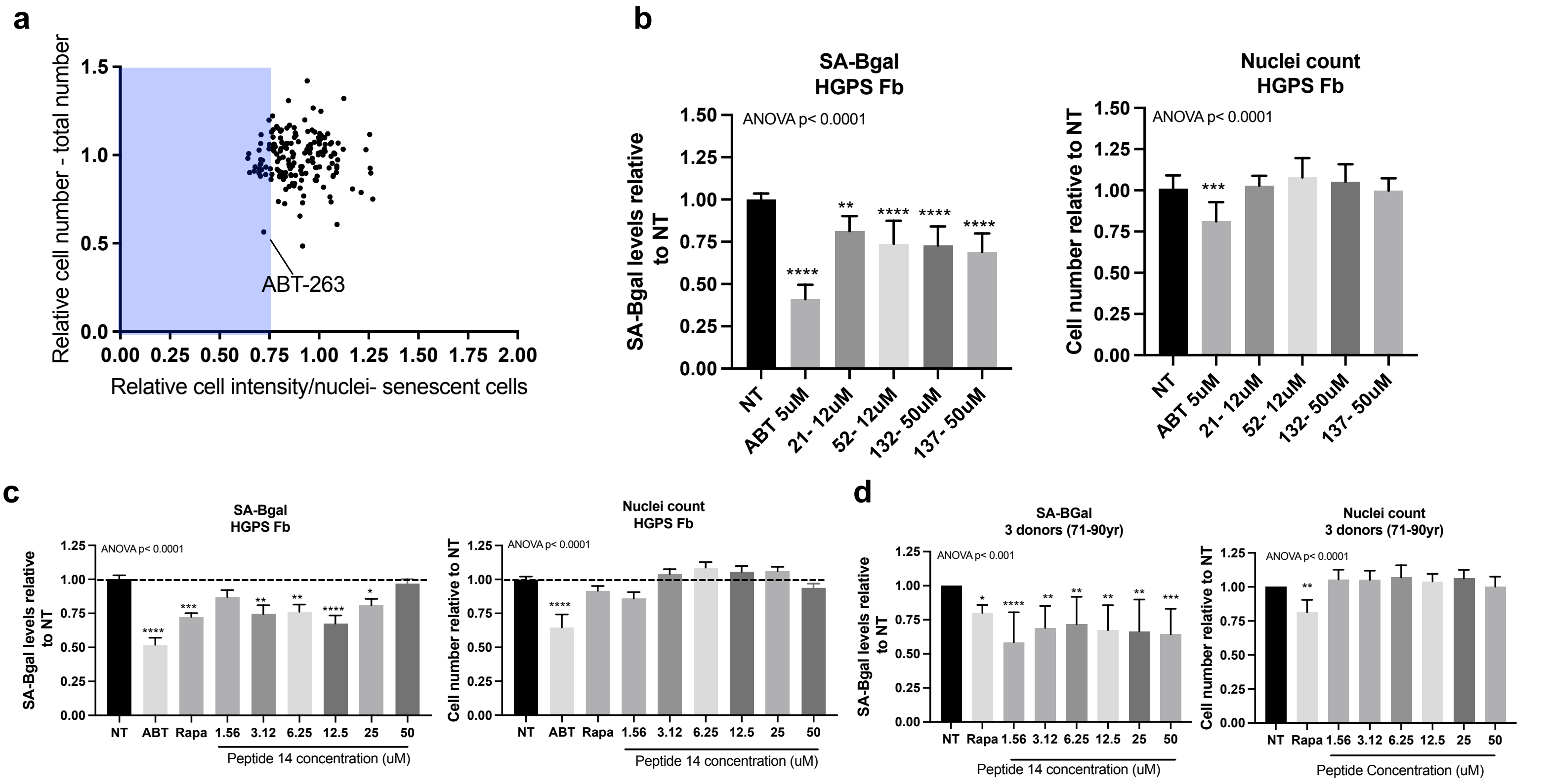

Supplementary Figure 2. Identification of new peptides with senotherapeutic properties. (a) Screening of 164-peptides according to the senotherapeutic potential. Peptides that reduced the number of senescent cells greater than 25% were considered senotherapeutic and highlighted in the blue shaded area. (b) Relative cellular senescence and cell count of HGPS HDFs treated for 48 hours with the top 4 peptides screened in (a), with ABT-263 (ABT) as control. (c) Relative cellular senescence and cell count of HGPS HDFs treated with different concentrations of Pep 14. (d) Relative cellular senescence and cell number of HDFs to non-treated controls obtained from 3 healthy donors with ages between 71 and 91 years and treated with different concentrations of Pep 14. $^{**}p<0.01$; $^{***}p<0.001$; $^{****}p<0.0001$, according to One-Way ANOVA and multiple comparisons. Data are shown as mean ± SD, n=3.

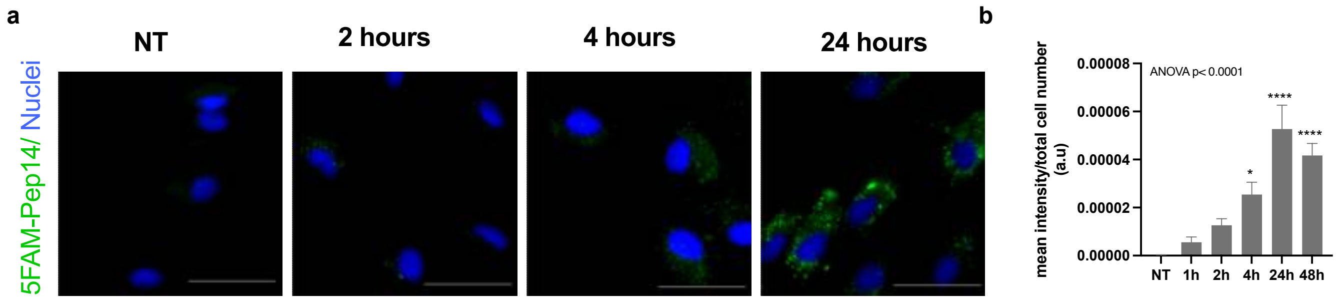

Supplementary Figure 3. Intracellular location of Pep14 as confirmed by fluorescence microscopy. (a) HGPS HDFs were incubated with 12.5μM 5FAM-Pep 14 for 1 to 48h. Bar corresponds to 100μm. (b) Quantification of 5FAM-Pep14 fluorescence within the cells (n=3, 4 images per replicate). Data are representative of 3 independent experiments in triplicate and bars represents means with SD. *p<0.05; ****p<0.0001, according to One-Way ANOVA and multiple comparisons.

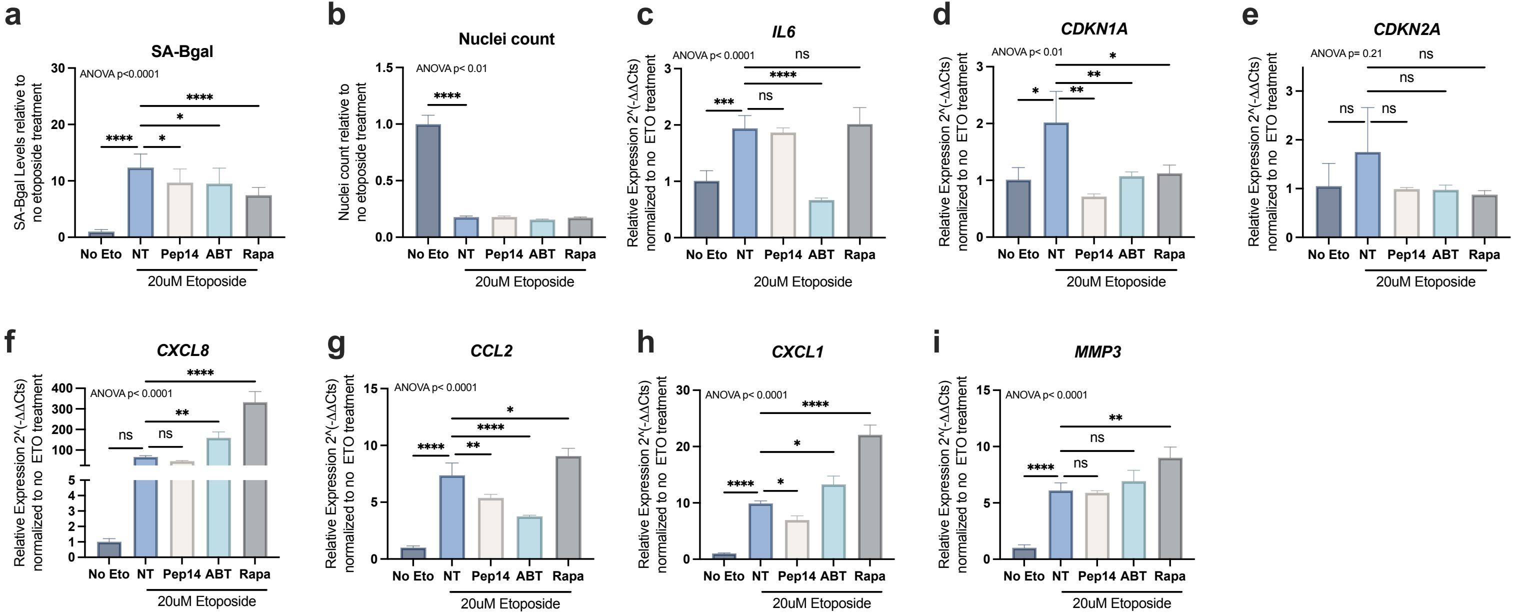

Supplementary Figure 4. Pep 14 protects cells from etoposide induced cellular senescence. HDFs were treated with 20 µM Etoposide for 24 hours, followed by 48 hours with fresh media and treated for additional 48 hours with 12.5 µM Pep 14, 5 µM ABT and 100 nM Rapamycin. (a) SA-βGal levels and (b) nuclei count relative to control cells that were not treated with etoposide (no eto). (c-i) mRNA gene expression relative to control cells not treated with etoposide. Data representative of 3 independent experiments in triplicate and bars represents means with SD. *p<0.05; **p<0.01; ***p<0.001; ****p<0.0001, compared to NT, according to one-way ANOVA and multiple comparisons test.

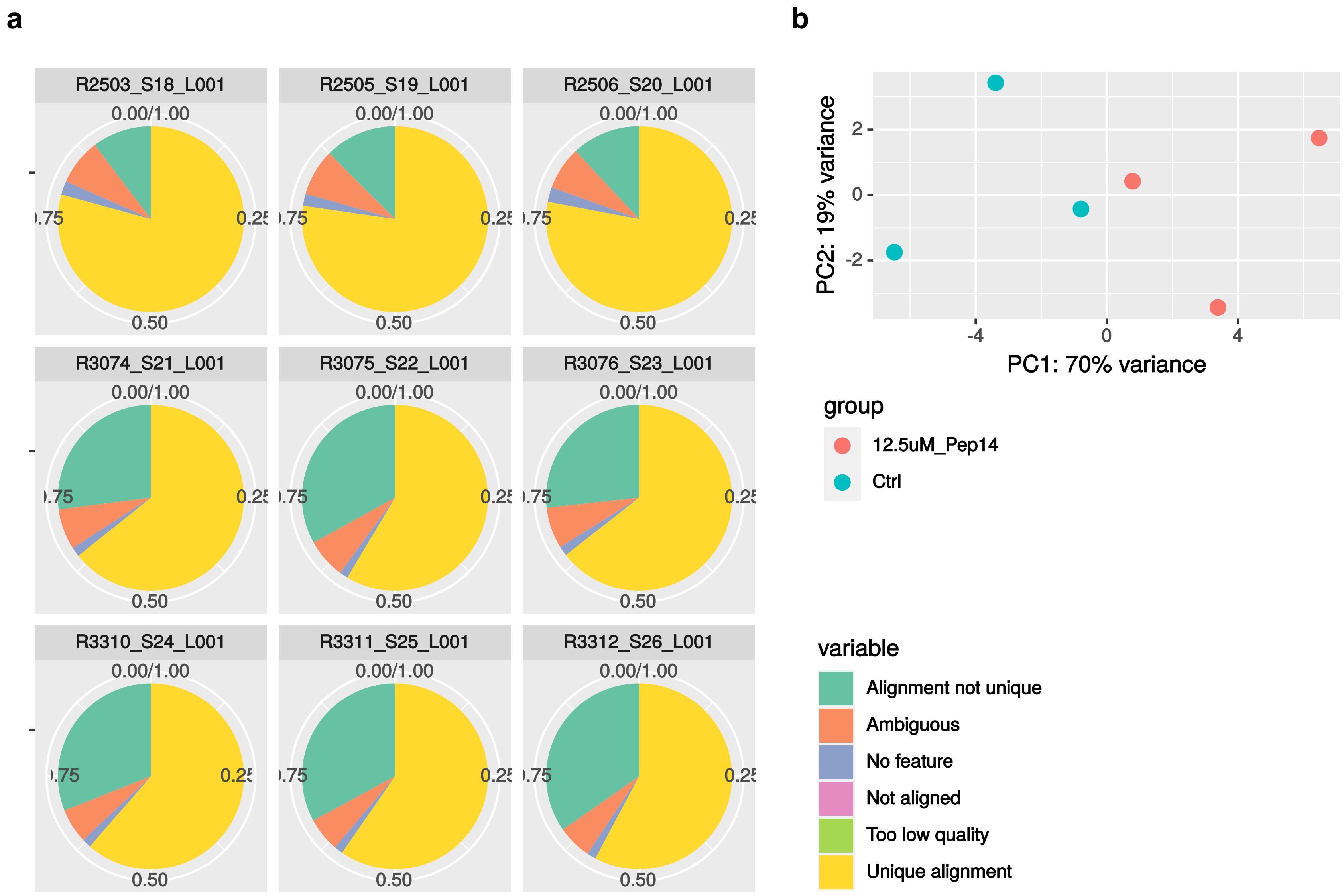

Supplementary Figure 5. RNA-Seq data characteristics. (a) Mapping quality analysis of reads derived from HDFs-HGPS RNA-Seq libraries. The description of each sample is depicted in the Supplementary Table 1. The graphs represent the percentage of reads mapped according to the color code. Only unique mapped reads (yellow) were considered for further analysis. We obtained an average of 70 million reads per sample. More than 1.2 billion good quality reads (corresponding to more than 95% of bases showing quality superior to Q30 in the Phred scale, with mean quality of Q36) were generated for all libraries (Supplementary Table 1). The majority of the reads (67%) were uniquely mapped with high quality to the human genome. (b) Principal component analysis (PCA) showing PC1 and PC2 for control and Pep 14-treated HGPS fibroblast samples.

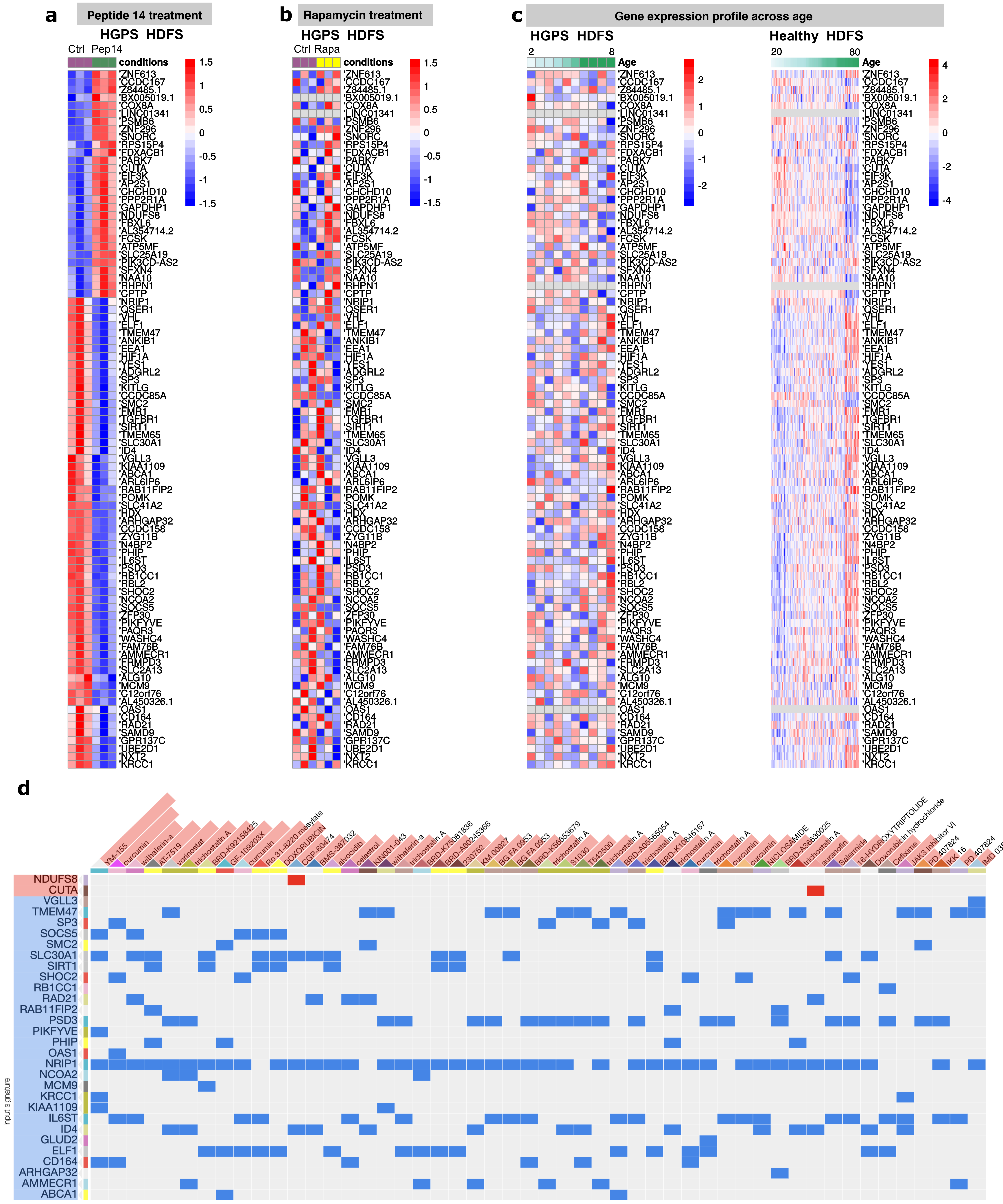

Supplementary Figure 6. Gene expression signature of top 89 genes modulated by Pep 14. (a) Heat maps showing the expression pattern of genes among different sample conditions for control and Pep 14-treated (12.5 μM) fibroblasts obtained from HGPS. To compare Pep 14-treated and control groups, samples were hierarchically clustered using distance as 1 − Pearson correlation coefficient. (b) The signature was also evaluated in control and Rapamycin-treated (100 nM) fibroblasts obtained from HGPS and in (c) HDFs samples derived from HGPS patient and healthy donors sorted according to the donor's age. Color codes represent RNA-seq normalized pseudocounts in log2 scale after row-wise z-score transformation. (d) Drugs that mimic the Pep 14 signature. Input (Pep 14) gene signatures are depicted as the rows of the matrix and the expression levels of the genes are indicated with red or blue label-bars (indicating up or down expression). The top-ranked L1000 perturbations with the most similar signatures to input are shown as columns with red label-bars that indicate their score. L1000 perturbation gene signatures are depicted as columns of the matrix with red and blue squares indicating their effect on gene expression.

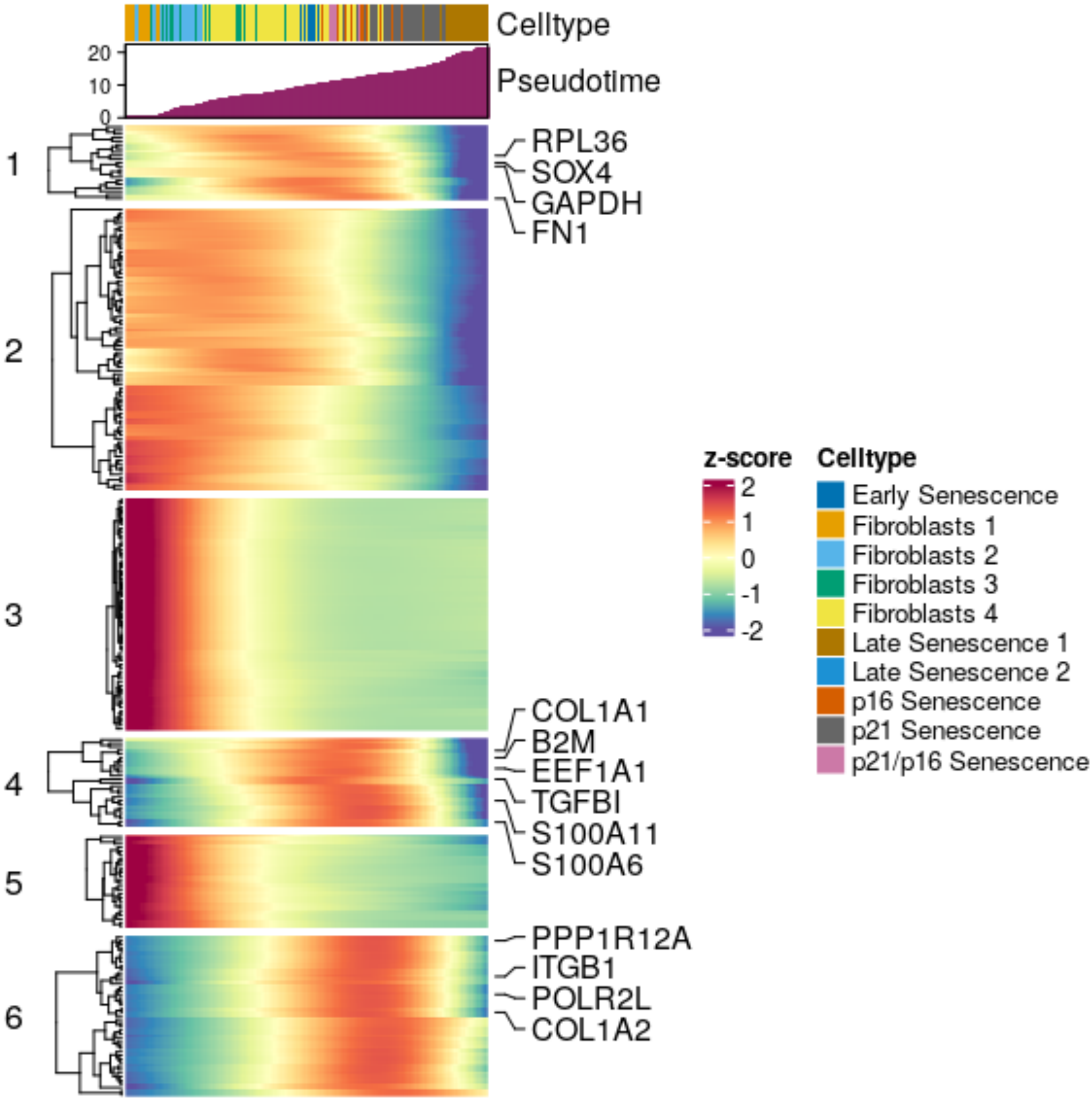

Supplementary Figure 7. Genes modulated during fibroblast senescence trajectory. Branched heatmap showing significant genes that covariate according to pseudotime. Genes are clustered according to their expression patterns (color coded through the z-score), and important genes in the senescence process have been highlighted. Cells are ordered according to their pseudotime in the differentiation process.

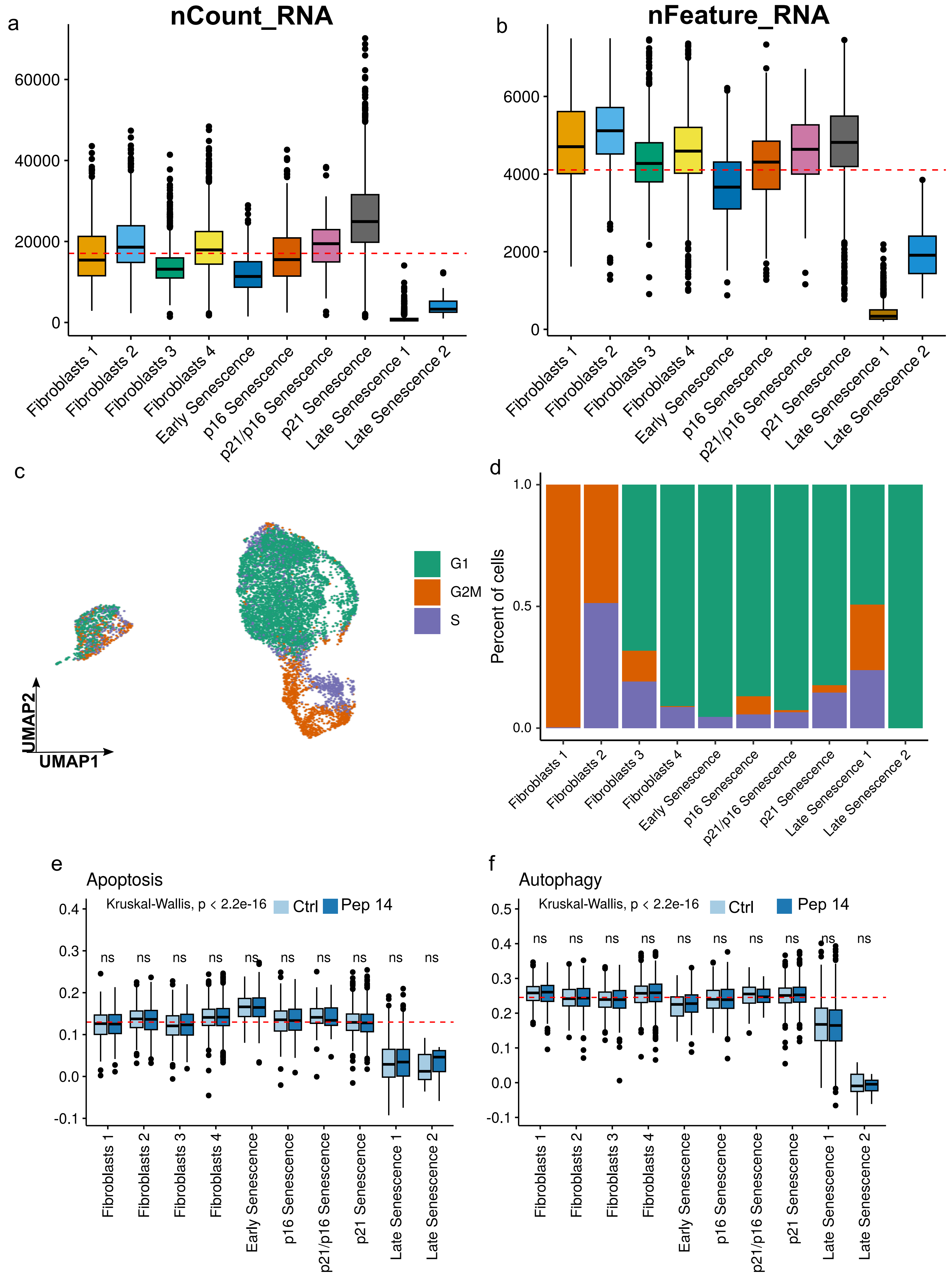

Supplementary Figure 8. Single-cell RNASeq. (a) Number of UMIs and (b) genes detected per cell in each cluster. Dashed lines in red represent the average values. Box indicates the range from 25th to 75th percentile, with whiskers extending to 1.5 times the interquartile range. Outliers are plotted separately, center indicates the median value. (c) UMAP represents the cell cycle stage of different cell populations identified in the HGPS dermal fibroblasts. (d) Percentage of cells in G1, G2M, and S in each cluster. (e) Score based on apoptosis and (f) autophagy expression in each cell cluster, with or without Pep 14 treatment. Dashed lines in red represent the average values. Differences in peptide versus control were compared by Wilcoxon test with Bonferroni correction (ns: non-significant). Box indicates the range from 25th to 75th percentile, with whiskers extending to 1.5 times the interquartile range. Outliers are plotted separately, center indicates the median value.

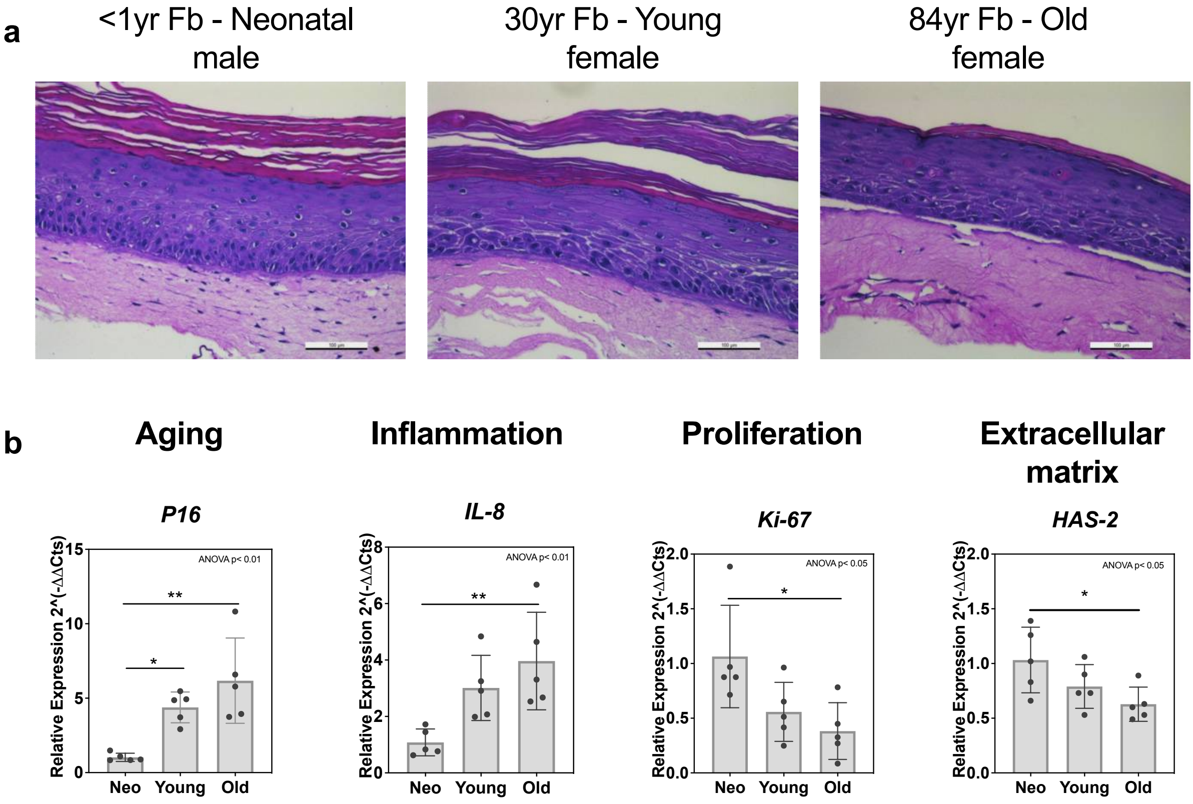

Supplementary Figure 9. Histological and molecular characterization of aged 3D skin equivalents. (a) Representative Hematoxylin and Eosin staining of histological sections of 3D skin equivalents built using HDFs obtained from <1 (neonatal, male), 30 (young, female), and 84 (old, female) year old donors. The histological scores of the 3D HSE, as analyzed by experimental group-blinded personnel are indicated as numbers below each image. (b) mRNA expression of aging, inflammation, proliferation and hyaluronic acid production markers in 3D skin equivalents built using cells obtained from Neonatal, young and old donor HDFs. **p<0.01, compared to the neonatal sample, according to one-way ANOVA followed by Bonferroni's multiple comparisons test. Data are shown as mean ± SD and dots represents independent experiments, n=5.

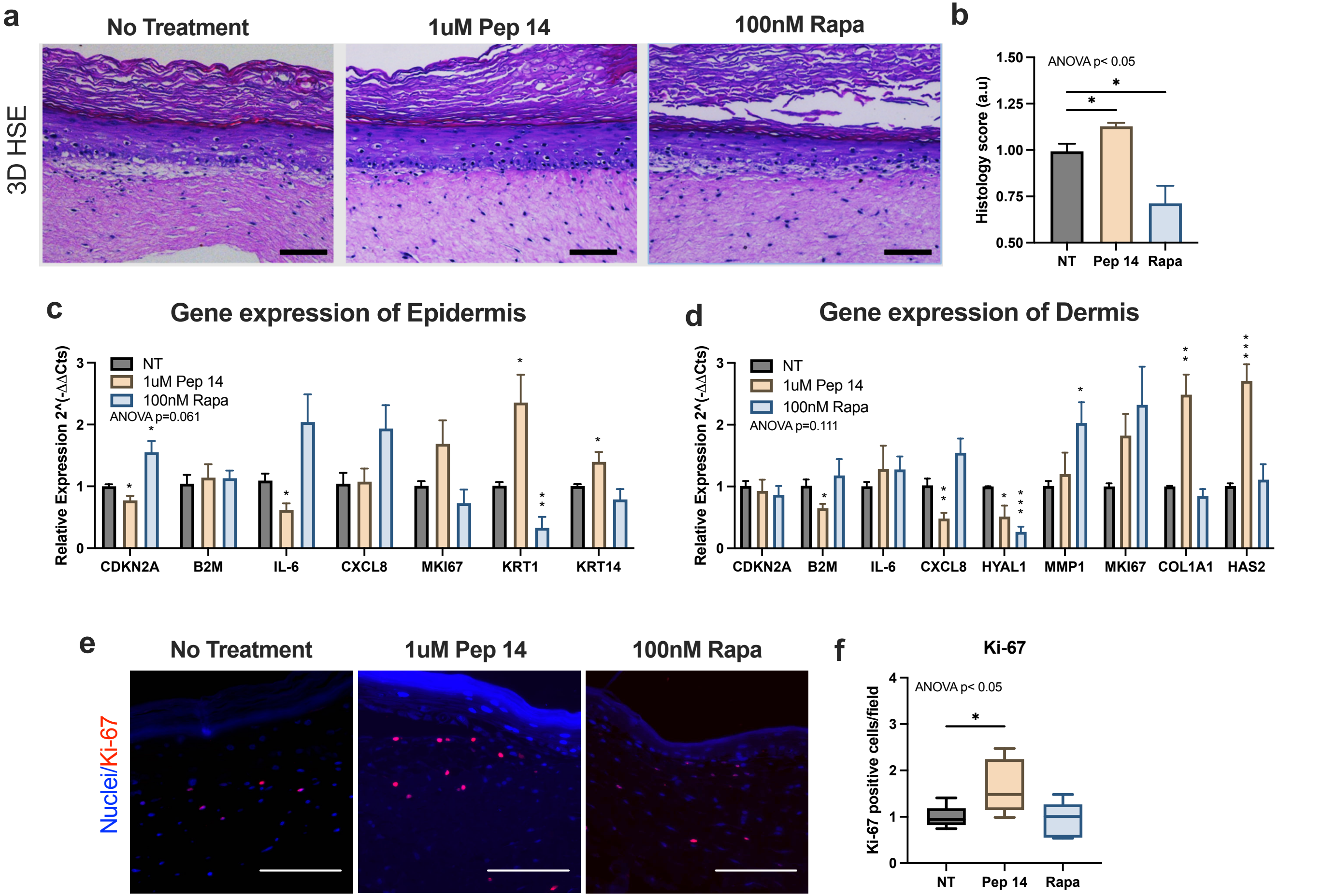

Supplementary Figure 10. Pep 14 treatment improves markers in three-dimensional human skin equivalents (HSE) built using cells of donors of different age ranges. HSE were built using primary cells from chronologically aged donors (32, 48 and 60yr, all female) and treated with basal media, 1 μM Pep 14 or 100 nM Rapamycin (Rapa) added in the media twice (day 0 and 3) for 5 days. (a) Representative Hematoxylin and Eosin staining of histological sections of control, Pep 14 and Rapamycin-treated 3D HSE (48y) after 5 days of starting treatment, scale bar 100 μm. (b) Histological scores of the 3D HSE, as analyzed by experimental group-blinded personnel. mRNA expression of the epidermal (c) and dermal layers (d) of control (NT), peptide 14 and Rapamycin-treated 3D HSE. (e) Fluorescence microscopy images of proliferative cells sustained with Ki-67. Scale bar corresponds to 100 μm. (f) Quantification of the percentage of Ki-67 positive cells. Graph bar data are shown as mean ± SD. Boxplot data are shown as median (centre line) and quartiles (1st and 3rd) and the minimum and maximum by the whiskers. Data representative of 3 independent experiments. *p<0.05; **p<0.01; ***p<0.001; ****p<0.0001, compared to untreated control (NT), according to one-way ANOVA and multiple comparisons.

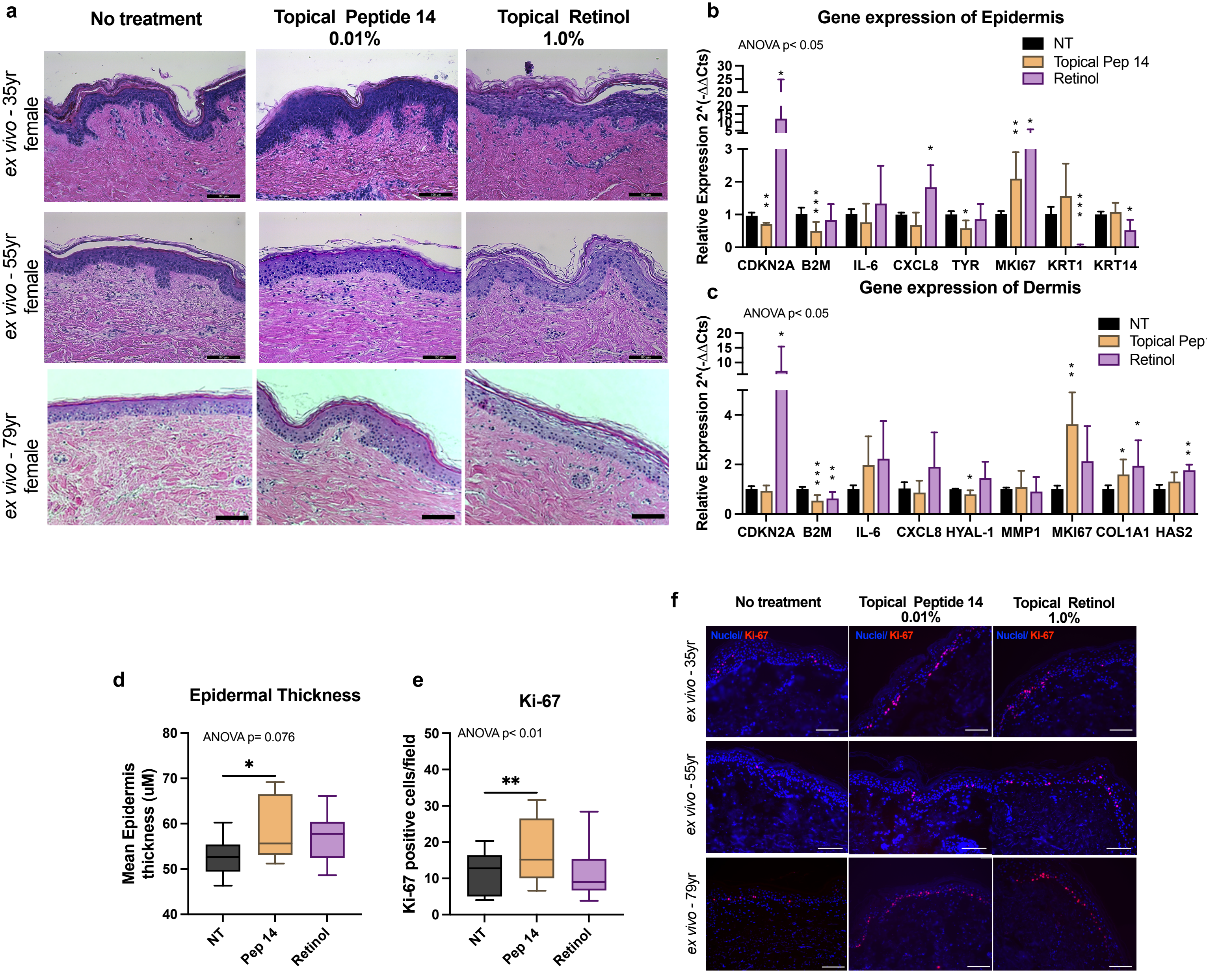

Supplementary Figure 11. Topical application of Pep 14 in ex vivo skin samples decreases cellular senescence and strengthens skin barrier. (a) Representative Hematoxylin and Eosin staining of histological sections of ex vivo skin samples from 35yr, 55yr and 79 yr donors (all female) maintained in basal media, or treated with topical Pep 14 or topical Retinol, scale bar 100 μm. (b) mRNA expression of epidermal and (c) dermal layers of treated samples (35, 55 and 79yr). (d) Epidermal thickness analysis of ex vivo skin samples (35, 55 and 79yr) maintained in basal media, or treated with topical Pep 14 or topical Retinol for 5 days. Quantification of the expression of Ki-67 (e) of ex vivo skin samples (35, 55 and 79yr). (f) Fluorescence microscopy representative images of ex vivo skins (35 yr, 55 yr and 79yr), 5 days after treatment, stained for Ki-67. Scale bar corresponds to 100 μm. Graph bar data are shown as mean ± SD. Boxplot data are shown as median (centre line) and quartiles (1st and 3rd) and the minimum and maximum by the whiskers. Data representative of 3 independent experiments in triplicate. *p<0.05; **p<0.01; ***p<0.001; compared to untreated control (NT), according to one-way ANOVA and multiple comparisons test or Student's t-test.

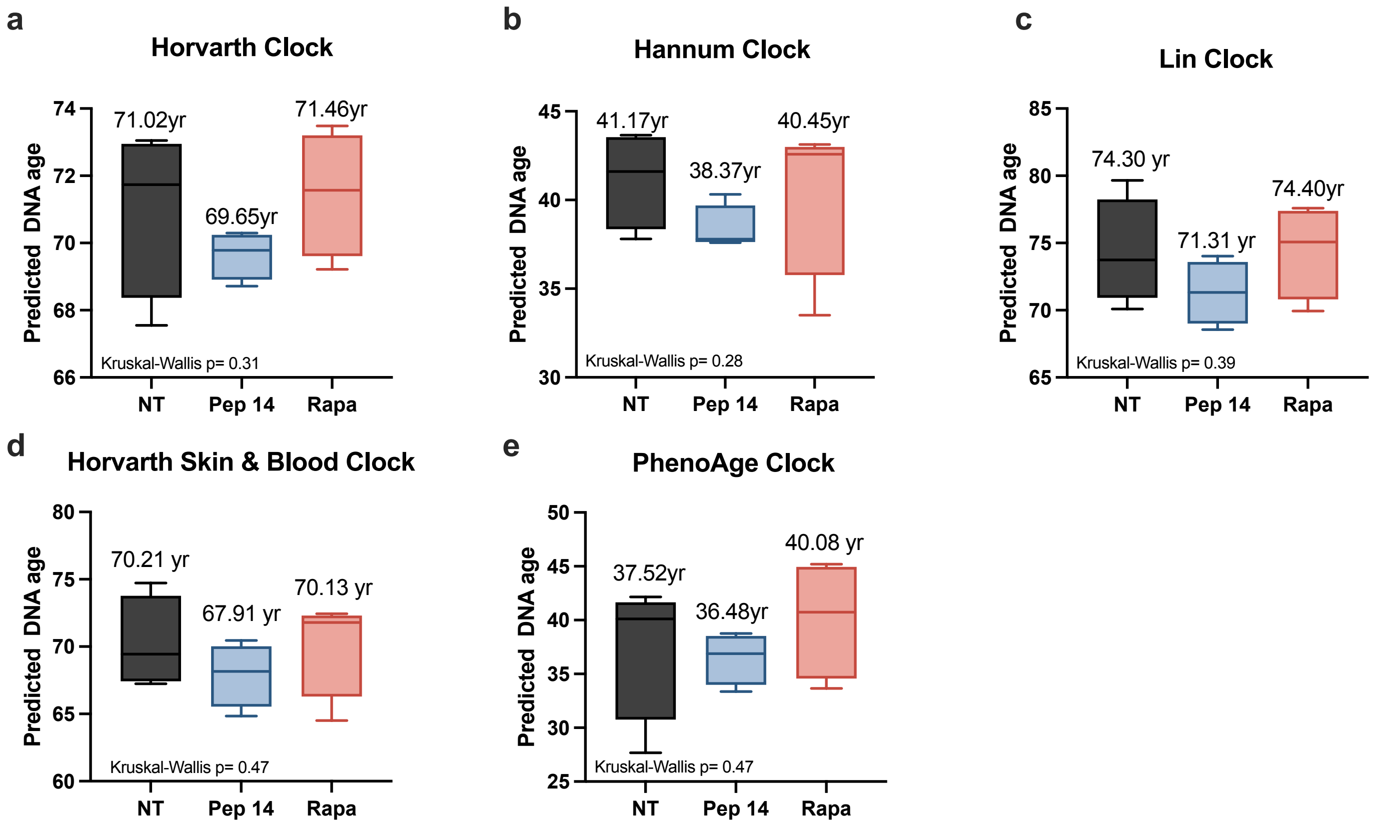

Supplementary Figure 12. DNA methylation age calculated using different epigenetic clocks. Ex vivo skin samples (79yr) maintained in basal media (NT), or treated with 100 nM Rapamycin (Rapa), or 12.5 μM Pep 14, added in the media for 5 days. Predicted DNA age according to Horvath (a), Hannum (b), Skin & Blood (c), PhenoAge (d) and Lin (e) epigetic clocks. Data representative of 1 experiment with 4 biological replicates, statistical analysis was done using Kruskal-wallis and Wilcoxon test. Boxplot data are shown as median (centre line) and quartiles (1st and 3rd) and the minimum and maximum by the whiskers.

Figure 1k, P16

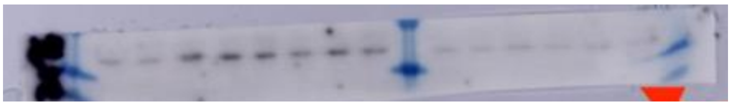

Figure 1k, B-actin loading control for P16

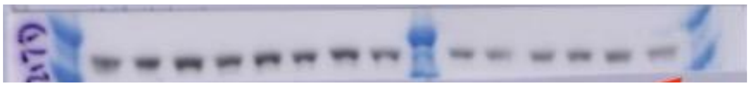

Figure 1k, yH2a.X

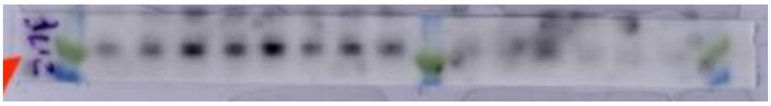

Figure 1k, B-actin loading control for H2A.x

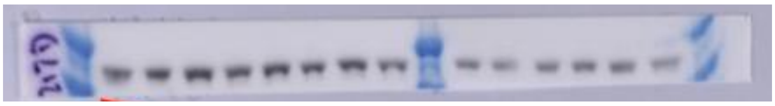

Figure 3e, PP21

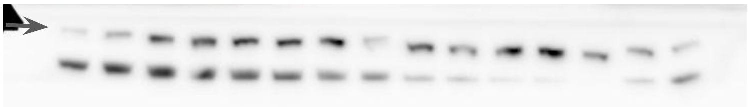

Figure 3e, Vinculin loading control for PP2A

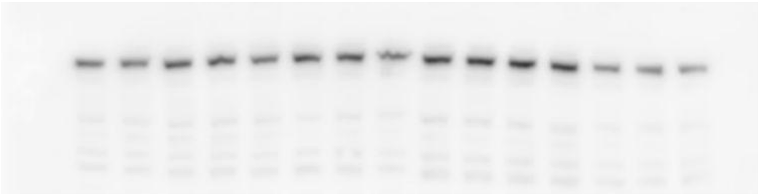

Figure 1k, P21

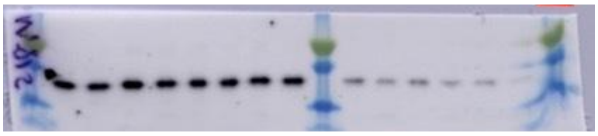

Figure 1k, B-actin loading control for P21

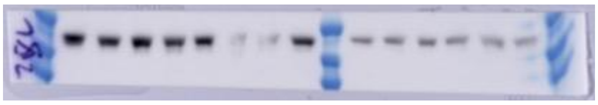

Figure 1k, H2A.X

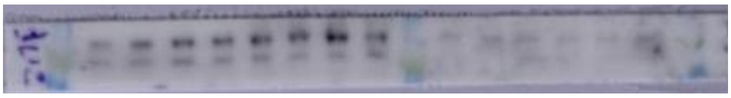

Figure 3f, p-AKT

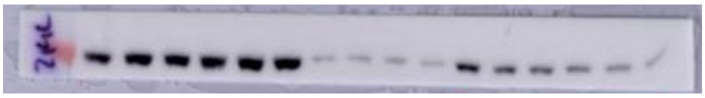

Figure 3f, p-AKT

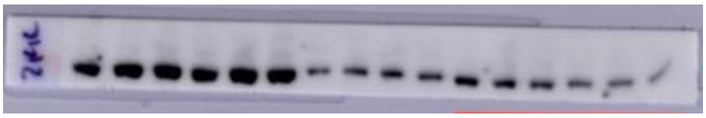

Figure 3f, B-Actin loading control for AKT

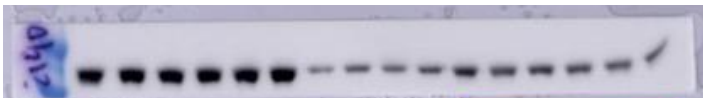

Supplementary Figure 13. Unprocessed Western blots. The original scans for the most important Western blots for Figure 1 and 3 are shown. Horizontal lines on top of the original scans mark the position of lanes that are relevant to the figure in the main text. Arrowhead mark the position of PP2A.

Supplementary Table 1 - Statistics and quality control of reads generated for each sample.

Sample_ID	Condition	Cell_type	Read Order	Index	Yield(Bases)	Reads	% of >= Q30 Bases(PF)	Mean Quality Score(PF)
R2503_S18_L001	Control	HGPS - Fibroblasts	R1	CCGCGGTT-AGCGCTAG	4143733565	41027065	96.66	36.47
R2503_S18_L001	Control	HGPS - Fibroblasts	R2	CCGCGGTT-AGCGCTAG	4143733565	41027065	95.41	36.24
R2505_S19_L001	48h with 12.5uM Pep14	HGPS - Fibroblasts	R1	TTATAACC-GATATCGA	3833050697	37950997	96.69	36.48
R2505_S19_L001	48h with 12.5uM Pep14	HGPS - Fibroblasts	R2	TTATAACC-GATATCGA	3833050697	37950997	95.49	36.26
R2506_S20_L001	48h with 100nM Rapa	HGPS - Fibroblasts	R1	GGACTTGG-CGCAGACG	3103306911	30725811	96.63	36.47
R2506_S20_L001	48h with 100nM Rapa	HGPS - Fibroblasts	R2	GGACTTGG-CGCAGACG	3103306911	30725811	96.19	36.39
R3074_S21_L001	Control	HGPS - Fibroblasts	R1	AAGTCCAA-TATGAGTA	3408218639	33744739	96.7	36.48
R3074_S21_L001	Control	HGPS - Fibroblasts	R2	AAGTCCAA-TATGAGTA	3408218639	33744739	95.73	36.31
R3075_S22_L001	48h with 12.5uM Pep14	HGPS - Fibroblasts	R1	ATCCACTG-AGGTGCGT	3467352523	34330223	96.58	36.46
R3075_S22_L001	48h with 12.5uM Pep14	HGPS - Fibroblasts	R2	ATCCACTG-AGGTGCGT	3467352523	34330223	95.42	36.25
R3076_S23_L001	48h with 100nM Rapa	HGPS - Fibroblasts	R1	GCTTGTCA-GAACATAC	3238628731	32065631	96.65	36.47
R3076_S23_L001	48h with 100nM Rapa	HGPS - Fibroblasts	R2	GCTTGTCA-GAACATAC	3238628731	32065631	95.39	36.24
R3310_S24_L001	Control	HGPS - Fibroblasts	R1	CAAGCTAG-ACATAGCG	3569528163	35341863	96.71	36.48
R3310_S24_L001	Control	HGPS - Fibroblasts	R2	CAAGCTAG-ACATAGCG	3569528163	35341863	95.68	36.29
R3311_S25_L001	48h with 12.5uM Pep14	HGPS - Fibroblasts	R1	TGGATCGA-GTGCGATA	3847078486	38089886	96.56	36.46
R3311_S25_L001	48h with 12.5uM Pep14	HGPS - Fibroblasts	R2	TGGATCGA-GTGCGATA	3847078486	38089886	95.84	36.32
R3312_S26_L001	48h with 100nM Rapa	HGPS - Fibroblasts	R1	AGTTCAGG-CCAACAGA	4034131193	39941893	96.65	36.47
R3312_S26_L001	48h with 100nM Rapa	HGPS - Fibroblasts	R2	AGTTCAGG-CCAACAGA	4034131193	39941893	95.66	36.29

**Supplementary Table 2** - Gene expression of the top 20 modulated genes by peptide 14.							
**ENSEMBLE ID**	**baseMean**	**log2FoldChange**	**lfcSE**	**stat**	**pvalue**	**padj**	**gene_names**
ENSG00000227953	11.21928	1.8972427	0.7786993	2.436426	0.01483322	0.9999969	LINC01341
ENSG00000170684	23.85127	0.9758074	0.4794204	2.03539	0.04181167	0.9999969	ZNF296
ENSG00000182600	39.96606	0.9296937	0.4340989	2.141664	0.03222057	0.9999969	SNORC
ENSG00000125454	79.78432	0.6218552	0.2672605	2.326775	0.01997724	0.9999969	SLC25A19
ENSG00000198937	444.34585	0.343145	0.1709773	2.006962	0.04475374	0.9999969	CCDC167
ENSG00000102030	1269.75163	0.2995919	0.1440676	2.079523	0.03756933	0.9999969	NAA10
ENSG00000110717	2395.27336	0.2642114	0.1295688	2.039158	0.04143421	0.9999969	NDUFS8
ENSG00000042753	5153.4849	0.2493979	0.1253112	1.990229	0.04656572	0.9999969	AP2S1
ENSG00000023287	1551.10317	-0.2887837	0.1432133	-2.016458	0.04375207	0.9999969	RB1CC1
ENSG00000103479	924.35889	-0.3006971	0.145837	-2.061872	0.03921995	0.9999969	RBL2
ENSG00000072401	520.65902	-0.3841887	0.194788	-1.972343	0.0485705	0.9999969	UBE2D1
ENSG00000134352	4473.63731	-0.4318719	0.2142688	-2.015561	0.0438459	0.9999969	IL6ST
ENSG00000096717	453.74763	-0.4322946	0.2134931	-2.024864	0.04288128	0.9999969	SIRT1
ENSG00000136051	1411.46672	-0.4488149	0.2225324	-2.016852	0.04371101	0.9999969	WASHC4
ENSG00000165029	659.30446	-0.4849387	0.2409369	-2.012721	0.04414399	0.9999969	ABCA1
ENSG00000101888	164.93542	-0.5077906	0.2248966	-2.257885	0.02395283	0.9999969	NXT2
ENSG00000163291	495.70246	-0.5114793	0.2100275	-2.435297	0.01487956	0.9999969	PAQR3
ENSG00000151229	167.13173	-0.6399983	0.2705317	-2.365706	0.01799574	0.9999969	SLC2A13
ENSG00000172201	225.08422	-0.6415893	0.3191862	-2.010079	0.04442288	0.9999969	ID4
ENSG00000185900	27.98257	-1.1651732	0.4940269	-2.358522	0.01834788	0.9999969	POMK

**Supplementary Table 3** - Pathway Enrichment analysis using the program Enrichr with the top 20 genes modulated by Peptide14 as query genes. The KEGG 2021 database was used.					
**Name**	**Genes**	**P-value**	**Adjusted p-value**	**Odds Ratio**	**Combined score**
Huntington disease	RB1CC1 | NDUFS8 | AP2S1	0.002441	0.06504	12.99	78.14
Longevity regulating pathway	SIRT1 | RB1CC1	0.003736	0.06504	24.85	138.92
Amyotrophic lateral sclerosis	RB1CC1 | NDUFS8 | NXT2	0.003982	0.06504	10.87	60.07
FoxO signaling pathway	SIRT1 | RBL2	0.006082	0.06959	19.24	98.16
Signaling pathways regulating pluripotency of stem cells	IL6ST | ID4	0.007206	0.06959	17.59	86.77
Cellular senescence	SIRT1 | RBL2	0.008521	0.06959	16.09	76.69

Supplementary Table 4 - Pathway Enrichment analysis using the program Enrichr with the top 89 genes modulated by Peptide14 as query genes. The KEGG 2021 database was used.

Name	Genes	P-value	Adjusted p-value	Odds Ratio	Combined score
Endocytosis	TGFBR1 | AP2S1 | RAB11FIP2 | PSD3 | EEA1	0.003786	0.2026	5.17	28.84
Amyotrophic lateral sclerosis	NDUFS8 | COX8A | RB1CC1 | PSMB6 | NXT2 | CHCHD10	0.003824	0.2026	4.31	24.01
TGF-beta signaling pathway	TGFBR1 | PPP2R1A | ID4	0.006811	0.2058	8.27	41.28
Huntington disease	NDUFS8 | COX8A | RB1CC1 | PSMB6 | NXT2 | CHCHD10 | AP2S1	0.008487	0.2058	4.23	20.18
Th17 cell differentiation	TGFBR1 | HIF1A | IL6ST	0.009709	0.2058	7.23	33.53
FoxO signaling pathway	TGFBR1 | SIRT1 | RBL2	0.0167	0.2694	5.87	24.03
Parkinson disease	NDUFS8 | COX8A | PSMB6 | PARK7	0.01925	0.2694	4.12	16.27
D-Glutamine and D-glutamate metabolism	GLUD2	0.02033	0.2694	61.46	239.43
Cellular senescence	TGFBR1 | SIRT1 | RBL2	0.02633	0.3102	4.91	17.84
Renal cell carcinoma	VHL | HIF1A	0.03263	0.3314	7.41	25

**Supplementary Table 5** - Peptide penetration					
**Sample ID**	**Compound**	**Matrix**	**Tissue Weight (mg)**	**Concentration in Dermis (ng)**	**Penetration (%)**
HS8A	Peptide_14	dermis sample	93.5	4.4319	2.21595
HS8B	Peptide_14	dermis sample	90.1	3.35172	1.67586
HS8C	Peptide_14	dermis sample	71.1	4.39398	2.19699
